# Robust estimation of cortical similarity networks from brain MRI

**DOI:** 10.1038/s41593-023-01376-7

**Published:** 2023-07-17

**Authors:** Isaac Sebenius, Jakob Seidlitz, Varun Warrier, Richard A. I. Bethlehem, Aaron Alexander-Bloch, Travis T. Mallard, Rafael Romero Garcia, Edward T. Bullmore, Sarah E. Morgan

**Affiliations:** 1 Department of Psychiatry, University of Cambridge, Cambridge, UK; 2 Department of Computer Science and Technology, University of Cambridge, Cambridge, UK; 3 Department of Psychiatry, University of Pennsylvania, Philadelphia, PA USA; 4 Department of Child and Adolescent Psychiatry and Behavioral Science, Children’s Hospital of Philadelphia, Philadelphia, PA USA; 5 Lifespan Brain Institute, Children’s Hospital of Philadelphia, Philadelphia, PA USA; 6 Autism Research Centre, Department of Psychiatry, University of Cambridge, Cambridge, UK; 7 Department of Psychiatry, Harvard Medical School, Boston, MA USA; 8 Psychiatric and Neurodevelopmental Genetics Unit, Center for Genomic Medicine, Massachusetts General Hospital, Boston, MA USA; 9 Instituto de Biomedicina de Sevilla (IBiS) HUVR/CSIC/Universidad de Sevilla/CIBERSAM, ISCIII, Dpto. de Fisiología Médica y Biofísica, Barcelona, Spain; 10 Alan Turing Institute, London, UK

**Keywords:** Network models, Genetics of the nervous system, Magnetic resonance imaging

## Abstract

Structural similarity is a growing focus for magnetic resonance imaging (MRI) of connectomes. Here we propose Morphometric INverse Divergence (MIND), a new method to estimate within-subject similarity between cortical areas based on the divergence between their multivariate distributions of multiple MRI features. Compared to the prior approach of morphometric similarity networks (MSNs) on *n* > 11,000 scans spanning three human datasets and one macaque dataset, MIND networks were more reliable, more consistent with cortical cytoarchitectonics and symmetry and more correlated with tract-tracing measures of axonal connectivity. MIND networks derived from human T1-weighted MRI were more sensitive to age-related changes than MSNs or networks derived by tractography of diffusion-weighted MRI. Gene co-expression between cortical areas was more strongly coupled to MIND networks than to MSNs or tractography. MIND network phenotypes were also more heritable, especially edges between structurally differentiated areas. MIND network analysis provides a biologically validated lens for cortical connectomics using readily available MRI data.

## Abstract (web summary)

Sebenius et al. present Morphometric INverse Divergence (MIND), a robust MRI-based metric of similarity between brain areas that reflects biological factors that define cortical network architecture, such as gene expression and axonal connectivity.

## Main

A single structural magnetic resonance imaging (MRI) scan of a human brain contains an immense amount of information. Standard MRI-based surface reconstructions of the cortex, for example, comprise hundreds of thousands of vertices, each characterized by many features or phenotypes^1^. The challenging task of integrating this wealth of information to model the structural architecture of the brain is essential for a better understanding of healthy and disordered brain development and function.

Traditional, univariate studies of brain structure focus on individual MRI features, such as cortical thickness (CT) or volume, with recent large-scale research in this vein mapping the developmental trajectories for each of multiple regional (cortical and subcortical) gray matter volumes^2^. However, brain regions do not function or develop in isolation but, instead, form an integrated, genetically coordinated, anatomically interconnected network. Accurately modeling the network architecture or connectome of the brain is crucial for understanding its putative role across typical and atypical functioning and development^3–5^. Recently, the construction of structural similarity networks has emerged as a promising approach for integrating multiple structural MRI features into biologically relevant single-subject connectomes^6,7^. Morphometric similarity networks (MSNs), the prototypical such method, are based on representing each brain region as a vector of several MRI features, typically including macrostructural metrics—for example, CT—as well as microstructural metrics—for example, the T1w/T2w ratio between longitudinal relaxation time (T1-) and transverse relaxation time (T2-) weighted data, a marker of cortical myelination. The morphometric similarity between regions is then estimated by the pairwise correlation between (standardized) regional feature vectors.

Although simple in construction, MSNs have demonstrated the promise of structural similarity networks to link macroscale MRI phenotypes with their neurobiological substrates. For example, MSNs recapitulated known brain organizational principles and cortical cytoarchitectonic classes^8^ more robustly than similar networks derived from tractography of diffusion-weighted imaging (DWI) data in *n* ~ 300 healthy young adults^6^. Moreover, MSNs from macaque MRI data were positively correlated with gold standard axonal connectivity measured by tract tracing^6^. Most promisingly, MSNs have provided a useful bridge between brain structure, cortical gene expression and genetics. For example, by combining cortical transcriptomic data from the Allen Human Brain Atlas (AHBA)^9^ with structural MRI from individuals with one of six different chromosomal copy number variation (CNV) disorders, Seidlitz et al.^10^ demonstrated that the changes in morphometric similarity induced by each CNV closely resembled the spatial patterning of expression of genes from the affected chromosome. Other studies have shown that changes in morphometric similarity in psychotic disorders^11^, major depressive disorder^12^ and Alzheimer’s disease^13^ correspond to the cortical expression of disease-relevant genes.

Despite the promise of MSNs, they suffer from two technical constraints: (1) they reduce the rich, vertex-level data from MRI-based cortical surface reconstructions to single summary statistics for each feature per region; and (2) their construction is based on standardized statistics (*z*-scores) that unrealistically force each MRI feature to be equally variable across cortical areas. Although other work has explored structural similarity measured directly from vertex-level data, these methods were limited to the use of a single structural feature, such as CT^14^ or gray matter volume^15,16^.

Here we propose Morphometric INverse Divergence (MIND) as a novel method for estimating structural similarity networks from MRI data. Each cortical area is characterized by a multidimensional distribution of multiple structural MRI features measured at each of many vertices—for example, vertex-wise measures of CT and curvature. The MIND similarity between each pair of regions is then derived from the symmetric Kullback–Leibler (KL) divergence (also known as Jeffrey’s divergence^17^) between their multivariate distributions.

Using more than 11,000 scans from three large human cohorts and one dataset of non-human primates, we compared MIND networks to MSNs and to networks derived by tractography of DWI data, across a suite of analyses designed to evaluate their relative performance against three major criteria, namely: (1) technical reliability, indexed by between-subject variability and resilience to noise; (2) biological validity, indexed by recapitulation of known anatomical principles of cortical organization, coupling with gene expression and genetic heritability; and (3) developmental sensitivity, indexed by prediction of age from individual differences in brain networks.

## Results

### MIND estimation

**Fig. 1:**
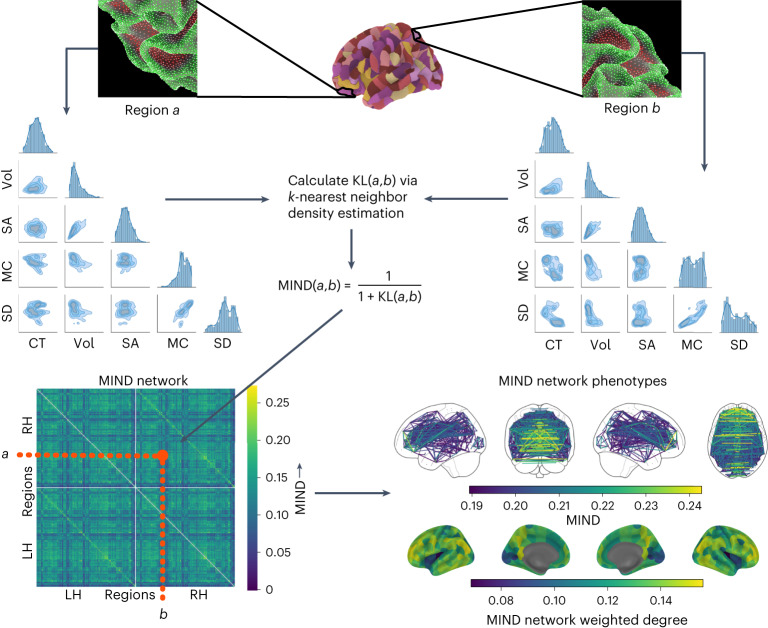
Estimation of MIND. As input, we used the mesh reconstructions of the cortical surface generated from T1w MRI scans by FreeSurfer’s recon-all command^48^. This surface can be described by a set of vertices (163,842 vertices per hemisphere for the fsaverage template^1^). Each vertex was characterized by five structural MRI features: CT, SA, Vol, MC and SD. To estimate the similarity between cortical areas, we standardized each MRI feature across all vertices and then aggregated all the MRI metrics for all vertices within each cortical area (defined by a prior parcellation template) to form a regional multivariate distribution. We then compiled a pairwise distance matrix using a *k*-nearest neighbor density algorithm to estimate the symmetrized KL divergence^49^, also known as Jeffrey’s divergence^17^, between each pair of regional multivariate distributions. Finally, we transformed the KL divergence *KL*(*a*,*b*) for regions *a* and *b* to estimate the inter-areal MIND similarity, bounded between 0 and 1, with higher values indicating greater similarity. Illustrative distributions for regions *a* and *b* are shown as scatter plot matrices, with diagonal panels showing the marginal univariate distribution for five structural features and the off-diagonals showing each pairwise bivariate relationship. Bottom row: visualization of a group mean MIND similarity matrix and cortical surface maps of two elementary MIND network phenotypes—that is, edges between cortical nodes (the top 2% are shown here) and weighted nodal degree, calculated as the average edge weight for each of 318 cortical nodes defined by the DK parcellation.

The pipeline for constructing MIND networks is summarized in Fig. 1 and Supplementary Fig. 1. A more rigorous definition of MIND as a similarity metric, in addition to a description of the *k*-nearest neighbor algorithm used to estimate symmetric multivariate KL divergence^18^, is provided in the Methods.

### Data and network construction

As our principal human MRI dataset, we used data from 10,367 individuals (aged 9–11 years) from the Adolescent Brain Cognitive Development (ABCD) study^19^, including 641 twin pairs. We also extended our analyses to the Human Connectome Project-Young Adult (HCP-YA, *n* = 960, aged 21–35 years) and Human Connectome Project-Development (HCP-D, *n* = 655, aged 8–21 years) cohorts^20,21^, two independent datasets comprising individuals of different age groups. For each individual, we constructed MSNs and MIND networks using a symmetric subdivision of the Desikan–Killiany (DK) atlas^22^ into 318 parcels of similar volume, henceforth referred to as DK-318 (ref. ^23^). We used the five morphometric features indicated in Fig. 1 for both MIND network and MSN construction: cortical thickness (CT), mean curvature (MC), sulcal depth (SD), surface area (SA) and gray matter volume (Vol), which was estimated at the vertex level by combining local measurements of thickness and area. These features are readily available from standard MRI processing pipelines using T1w images alone^1^; as such, we ensured that the method is applicable to most legacy structural MRI data. Details on the sensitivity of similarity network analysis to the choice of features can be found in Supplementary Fig. 6, and a comparison of group-level networks across cohorts is provided in Supplementary Fig. 14.

In the HCP-YA dataset, we also compared multivariate MIND networks to published connectomes derived by tractography of DWI data^24,25^ and to univariate MIND networks based on CT alone (Methods). Finally, we additionally accessed open gene expression data from the AHBA^9,26^, published macaque tract-tracing connectomes^27,28^ and MRI data from *n* = 19 macaques^29,30^. The macaque MRI included the same five structural features as for human data plus the T1w/T2w ratio as an estimate of intra-cortical myelination.

### Network reliability

We evaluated the technical reliability of MIND networks and MSNs as measures of brain network organization by examining the consistency of each method between subjects and measuring their dependence on the choice of parcellation template. We also evaluated the effect of including uninformative (noise) features into both types of network construction.

#### Between-subject consistency

**Fig. 2:**
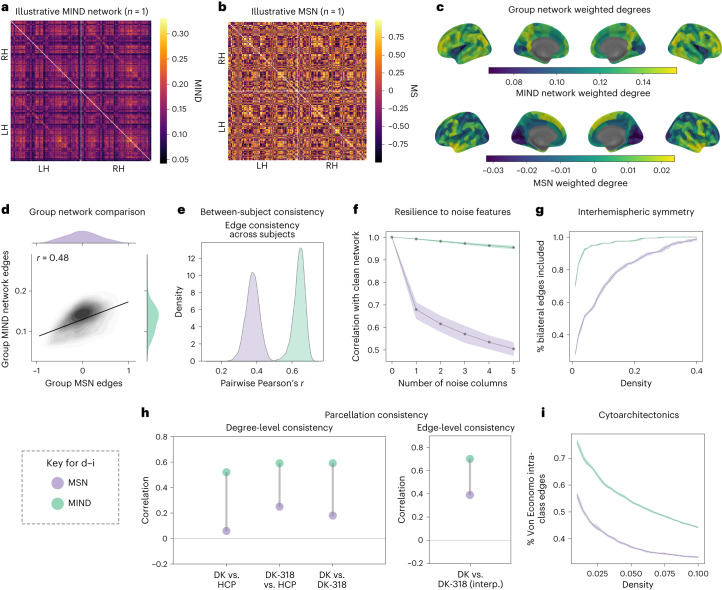
Cortical similarity connectomes: MIND networks and MSNs compared. **a**,**b**, Illustrative MIND network and MSN from the same randomly sampled participant in the ABCD cohort. LH, left hemisphere; RH, right hemisphere; MS, morphometric similarity. **c**, Cortical surface maps of group mean weighted degree for the MIND networks and MSNs. **d**, Scatter plot representing the positive correlation between edge weights of the group mean MIND networks and MSNs. **e**, The distributions of pairwise correlations of network edges between subjects for MIND networks and MSNs, for all pairs of 10,367 subjects. **f**, The correlation between MIND networks and MSNs constructed using 1–5 additional random features of Gaussian noise (for *n* = 150 random subjects). The solid line represents mean values, with shading representing empirical 95% confidence interval (CI). **g**, The fraction of total inter-hemispheric connections represented at different network densities for both group mean MIND networks and MSNs. **h**, Parcellation consistency of MIND network phenotypes at nodal level (weighted degree) and at edge level. The left plot shows the correlation between weighted degree estimated by each of the possible pairs of three parcellation templates: DK, DK-318 and HCP. To calculate between-parcellation correlations, each vertex was assigned the weighted degree of the region within which it was located, for each parcellation, and the correlation was calculated between the resulting vectors of vertex-wise values. The right plot shows the correlation between 2,278 network edges calculated using the 68-region DK parcellation or by using the finer-grained DK-318 parcellation to estimate 50,403 edges and coarse graining (DK-318 interp.) to match the number of edges in the original DK network. **i**, The fraction of edges between two regional nodes of the same cytoarchitectonic class over a range of network densities. In **g** and **i**, shading represents the 95% CI estimated by population bootstrapping, and the solid line represents the mean over all bootstrapped results. In all panels, except as noted in **h**, the DK-318 parcellation was used to define 318 cortical regions of approximately equal volume.

The group-level MSN and MIND networks were correlated in terms of both edge weights (*r* = 0.48; Fig. 2d) and weighted nodal degrees (*r* = 0.38). However, MIND networks were substantially more consistent across subjects (Fig. 2e), measured by pairwise correlation of edges (mean pairwise *r* = 0.62 versus *r* = 0.38) and degrees (mean pairwise *r* = 0.73 versus *r* = 0.45), suggesting that MIND network construction may lead to less noisy estimates of a common structural architecture. These results were replicated in the HCP-YA cohort, where multivariate (five-feature) MIND networks also showed increased inter-individual consistency compared to DWI tractography and univariate (CT-based) MIND networks (Supplementary Fig. 8).

#### Parcellation consistency

Brain network analysis assumes that major topological features can be replicated across cortical parcellations, and network-derived metrics should demonstrate high spatial consistency across parcellation schemes. We analyzed the consistency of group-level MSNs and MIND networks across three commonly used cortical parcellations of varied granularity: the 68-region DK atlas, the 318-region DK-318 atlas derived by subdivision of DK areas (the principal parcellation used for this study) and the 360-region HCP parcellation^31^.

We examined edge-level consistency by leveraging the fact that DK-318 is a strict subdivision of the DK atlas, allowing us to compare the original group DK networks with interpolated versions derived from the DK-318 group networks (Methods). MIND networks showed markedly higher edge consistency (Fig. 2h) in terms of the correlation between the original and interpolated DK networks (*r* = 0.70 versus *r* = 0.39 for MSNs).

To calculate between-parcellation correlations, each vertex was labeled by the weighted degree of the region to which it was assigned, for each parcellation, and the correlation was estimated between these two identical-length vectors of parcellation-specific degree projected to each vertex (Fig. 2h and Supplementary Fig. 5). MIND networks were strongly correlated across all (three) possible pairs of the three parcellations, whereas MSN degree demonstrated limited generalizability across parcellations (for example, *r* = 0.59 versus *r* = 0.18 for MIND networks and MSNs, respectively, when comparing weighted degree for DK and DK-318 atlases). We replicated these results in the HCP-YA dataset; here, univariate CT-based MIND networks demonstrated similarly high parcellation consistency, suggesting that the relative invariance to parcellation demonstrated by MIND networks over MSNs was due primarily to their use of vertex-level data (Supplementary Fig. 8).

#### Resilience to noisy features

We studied the robustness of MIND networks and MSNs to the inclusion of uninformative (noise) features. We created additional MIND networks and MSNs with between one and five ${{{\mathcal{N}}}}(0,1)$ noise features at each vertex (in addition to the five measured MRI features) for a random subset of 150 subjects. Because we standardized each morphometric feature, the non-random, measured variables also had a mean of 0 and a variance of 1. MIND networks constructed from these noisy data were almost perfectly correlated with MIND networks constructed from the measured features only (Fig. 2f), whereas MSN construction was substantially degraded by the inclusion of noise features (for example, mean *r* = 0.95 versus *r* = 0.50 for MIND networks and MSNs with five noise features).

### Validation by principles of cortical organization

We studied the extent to which each network type represented foundational principles known to govern cortical organization. Specifically, we benchmarked the biological validity of each type of structural similarity using the following basic premises about four known principles of brain structure:

- Symmetry: The cortex is highly symmetric, and homologous regions of right and left hemispheres are reciprocally interconnected, so a valid measure of structural similarity should have strong weights for inter-hemispheric edges while respecting known structural asymmetries.
- Cortical microstructure: Cortical areas can be cytoarchitectonically classified based on microstructural properties measured histologically, so a valid MRI measure of structural similarity should have strong weights for edges between cortical areas histologically assigned to the same cytoarchitectonic class^8^.
- Axonal connectivity: Cortical areas are interconnected by white matter tracts, and cytoarchitectonically similar regions are more likely to be axonally interconnected^32^, so a valid measure of structural similarity should correlate with axonal connectivity as measured by gold standard tract tracing in non-human primates.
- Developmental remodeling: The cortex undergoes substantial, coordinated remodeling across the lifespan^2^, so a valid measure of structural similarity should accurately detect developmental changes in the brain.

#### Symmetry and inter-hemispheric connections

Across a range of network densities, we measured how many bilateral connections were represented by each type of group mean network. Over all densities, MIND networks comprised a substantially larger fraction of bilaterally symmetric connections than MSNs (Fig. 2g). This result was replicated in the HCP-YA cohort using two parcellations (Supplementary Fig. 8). Multivariate MIND networks also captured stronger inter-hemispheric connections than DWI-based tractography or univariate MIND networks. Moreover, inter-hemispheric MIND connections were more closely aligned than MSNs with known patterns of asymmetry of the SA of bilaterally homologous cortical areas (Supplementary Fig. 4).

#### Cytoarchitectonics and within-class connections

Next, we analyzed the extent to which MIND networks and MSNs recapitulated known patterns of cortical microstructure, measured by higher similarity between regions of the same Von Economo cytoarchitectonic class^8^. MIND networks demonstrated higher intraclass connectivity across a range of network densities (Fig. 2i), indicating a closer correspondence with known patterns of cytoarchitectonic similarity at the scale of neuronal organization. This result was replicated using two parcellations in the HCP-YA cohort, where multivariate MIND networks, but not MSNs, also demonstrated stronger within-class connections than DWI tractography or univariate MIND networks (Supplementary Fig. 8).

#### Axonal connectivity and structural similarity

Previous work showed that regions with similar cytoarchitecture are more likely to be connected by axonal tracts than regions that are microstructurally dissimilar^32–34^. We therefore anticipated that more robust estimation of structural similarity via MIND networks, compared to MSNs, would result in stronger correlations with axonal connectivity measured by retrograde tract tracing in the macaque monkey brain.

**Fig. 3:**
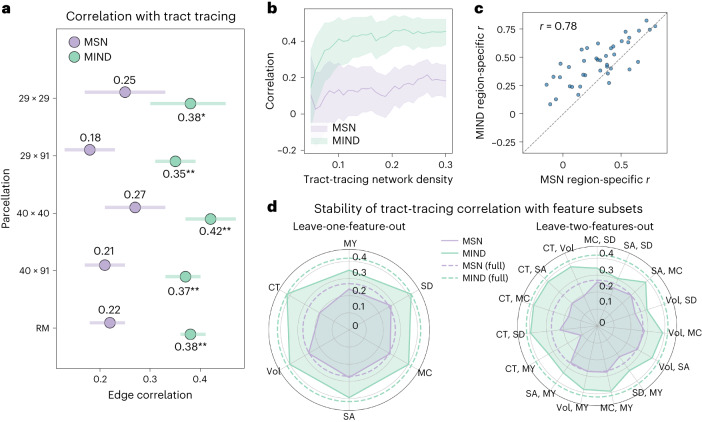
Structural similarity from MRI compared to axonal connectivity from tract tracing in the macaque brain. **a**, Correlation between structural similarity edge weights, in MIND networks or MSNs derived from macaque MRI, and axonal connectivity edge weights derived from tract tracing in five connectomes: the {29 × 29}, {29 × 91}, {40 × 40} and {40 × 91} versions of the Markov parcellation, with the number of target and source regions, respectively, indicated in each case^27,28^, and the whole-cortex connectome based on the separate RM parcellation^27,28,50^. The five connectomes contained *n* = 536, *n* = 1,615, *n* = 978, *n* = 2,229 and *n* = 3,267 edges, respectively. Shading indicates 95% confidence interval (CI). Asterisks indicate significantly increased correlation with tract-tracing data for MIND networks compared to MSNs, determined by bootstrapping network edges and performing a two-sided test on the difference in tract-tracing correlations: *P = 0.0018 and **P < 0.001, uncorrected. **b**, Correlation between tract-tracing {40 × 40} weights and MIND network or MSN edge weights over a range of tract-tracing network densities. Shading represents 95% CI. **c**, Scatter plot of the correlations between tract-tracing weights and MRI similarities (MIND or MS) for the set of edges connecting each regional node to the rest of the connectomes; thus, each point represents the correspondence between tract-tracing weights and structural similarity for each region in the {40 × 40} connectome (averaged for afferent and efferent connections; see Methods for details). The dashed line *y* = *x* highlights that similarities estimated by MIND were generally more strongly correlated with tract-tracing weights (above the line of identity) than morphometric similarities. **d**, Radar plots of the stability of the correlation between axonal connectivity, again from the {40 × 40} connectome, and structural similarity from MSNs or MIND networks, estimated over all possible input feature sets with one or two missing features. Missing features are noted at each radial position, with the radius from the center indicating correlation with tract-tracing weights. Best-case correlations for each type of structural similarity network estimated using all six MRI features are shown as dashed lines: MY, myelination (T1/T2 ratio).

Using MRI data from 19 macaques^29,30^, we constructed group-level MSN and MIND networks using the same five structural features as for human MRI analysis as well as the T1w/T2w ratio. We compared the correspondence between axonal connectivity and structural similarity across five tract-tracing connectomes based on two distinct cortical parcellations (detailed in Fig. 3a and Methods).

Replicating and extending the work by Seidlitz et al.^6^, which used a different macaque MRI dataset, we found that edge weights of axonal connectivity estimated from tract-tracing data were positively correlated with the corresponding edge weights of structural similarity estimated from MRI data by MSN or MIND network analysis (Fig. 3a). Axonal connectivity weights were significantly more positively correlated with MIND network edges than with MSN edges across all five connectomes analyzed (*P* < 0.01 from edge bootstrapping, Bonferroni corrected). Using the {40 × 40} matrix (the largest weighted connectome with complete source and target data), we recapitulated this result over a range of tract-tracing network densities (Fig. 3b). Moreover, the degree to which regional profiles of MIND and MS corresponded to a region’s tract-tracing connections was highly correlated (*r* = 0.78), although MIND showed a higher correspondence with regional tract tracing for 85% of regions (Fig. 3c).

To test the contribution of individual morphometric features, we recalculated the correlations between the {40 × 40} tract-tracing connectome and structural similarity networks estimated with all possible subsets of four or five (of the total set of six) MRI features. The greater positive correlation of tract tracing with MIND networks, compared to MSNs, was maintained across all feature subsets (Fig. 3d). Further analysis demonstrated that univariate MIND networks calculated with any single morphometric feature alone had reduced correspondence to tract-tracing networks, pointing to the importance of a multivariate approach (Supplementary Fig. 13).

#### Sensitivity to developmental changes

We gauged the sensitivity of MIND networks and MSNs to detect developmentally relevant inter-individual variation by comparison on the task of age prediction from brain MRI data in the HCP-D (ages 8–21 years) and HCP-YA (ages 21–35 years) cohorts. For HCP-YA, we also benchmarked both methods against DWI tractography^24^. Using either nodal degree or network edge weights as input, we trained machine learning models to predict each participant’s age, evaluating model performance over 10 data splits and controlling for several potential confounds (Methods).

**Fig. 4:**
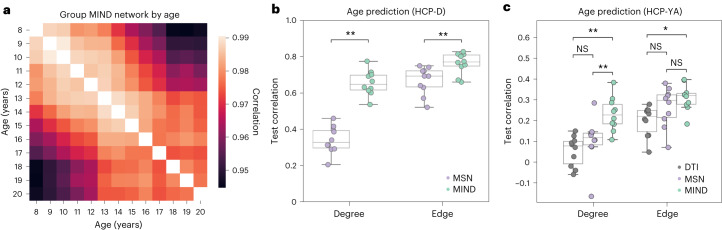
Predicting age from structural similarity and DWI tractography human brain networks. **a**, Pairwise correlation between the edges of age-specific MIND networks, computed by averaging over subjects grouped by age in years. All age-specific group-level networks were highly correlated (*r* > 0.94) but nonetheless demonstrated a clear age-dependent progression—that is, age-specific group networks became less similar when compared across larger age gaps. **b**, Comparison of the performance of models trained to predict age using nodal weighted degree or edge weights of either MIND networks or MSNs in the HCP-D cohort (ages 8–21 years). The dataset was split into 10 training and test sets (90:10 ratio, all test sets non-overlapping); each point indicates the performance on one test set. The *y* axis indicates the partial Spearman correlation between predicted and true age, corrected for sex, Euler index (a proxy for scan quality) and a global connectivity coefficient (the sum over all matrix elements) to mitigate confound effects. Further training and evaluation details are provided in Methods. Significantly differential performance between MSNs and MIND networks (**P < 0.01, FDR corrected from paired two-sided *t*-tests) was observed for both edge-based (*P* = 0.004) and degree-based (*P* = 0.004) models. **c**, An analogous plot to **b**, comparing the performance of models trained on MIND networks, MSNs or DWI tractography connectomes in the HCP-YA cohort (ages 21–35 years). Networks in **b** used the DK-318 parcellation, whereas networks in **c** were based on the HCP 360-region parcellation to match the publicly available DWI tractography connectomes (**P < 0.01 and *P < 0.05, FDR corrected from paired two-sided *t*-tests). Exact *P* values for edge-based models were as follows: MSN versus DTI (*P* = 0.11), MSN versus MIND (*P* = 0.12) and DTI versus MIND (*P* = 0.01). Exact *P* values for degree-based models were as follows: MSN versus DTI (*P* = 0.25), MSN versus MIND (*P* = 0.008) and DTI versus MIND (*P* = 0.002). For **b** and **c**, box plots indicate data quartiles, and whiskers indicate the full data range, excluding outliers. NS, not significant.

Predictive performances are summarized in Fig. 4. All models improved when trained on all network edges, reflecting information loss when considering node degree alone. Models trained on MIND degree outperformed other degree-based models in both datasets (for example, mean correlation with HCP-D test sets = 0.65 versus 0.34 for MIND and MS, respectively). Models trained on MIND network edges again showed the highest performance, although to a lesser extent (for example, mean correlation with HCP-YA test sets = 0.31, 0.27 and 0.20 for MIND, MS and DWI tractography, respectively). DWI tractography connectomes processed through a separate pipeline and using an alternative measure of connectivity^25^ gave highly consistent results (Supplementary Fig. 9 and Supplementary Table 1).

### Transcriptional similarity and structural similarity networks

The finding that morphometric similarity networks are spatially co-located with transcriptional similarity or gene co-expression networks^6^ builds on foundational work in imaging transcriptomics^35^ and has spurred subsequent research efforts to link MRI-derived connectomes to underlying transcriptional patterns^10,11,13,36,37^.

Following standardized processing protocols^38^, we combined high-resolution spatial gene expression data on six postmortem adult donors from the AHBA to generate an expression matrix for 15,633 genes in 34 regions from the left hemisphere of the DK atlas^9,26^. We then calculated the pairwise similarity of regional expression profiles to generate a {34 × 34} matrix of transcriptional similarity.

**Fig. 5:**
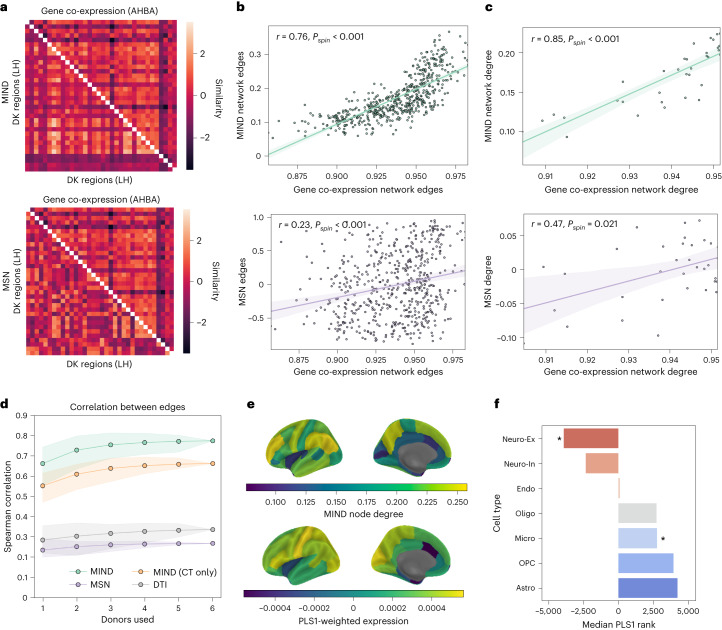
Structural similarity and transcriptional co-expression networks. **a**, Gene co-expression networks (upper triangles) compared to MIND networks and MSNs (lower triangles). LH, left hemisphere. **b**,**c**, Correlation between the gene similarity network and the group MSN or MIND network, at the level of edges (**b**) and weighted nodal degrees (**c**). Shading represents 95% confidence interval (CI) of best-fit line. *P* values (uncorrected) were based on a two-sided spin test that generated a distribution of null correlations from random spatial network rotations (Methods). **d**, Stability of the correlation between structural and transcriptomic networks constructed from all subsets of the six postmortem brain gene expression datasets available. Also included are results on univariate MIND networks derived from cortical thickness and consensus DTI connectivity from the HCP-YA dataset^39^. For each number of donors included, all combinations of transcriptional networks were constructed (without gene filtering), and the mean edge correlation was calculated for each network type. There were $$\begin{array}{l}\left(\begin{array}{l}6 \\ n \end{array}\right)\end{array}$$ possible networks created for *n* = 1, 2…6 included donors. Shading indicates the minimum and maximum value of the association observed for each number of included donors. **e**, Cortical brain maps of MIND weighted node degree beside a weighted gene expression map derived from PLS analysis of the covariation between degree, or ‘hubness’, of MIND nodes and gene expression. PLS1 explained a significant amount of covariance (62%, *P*_*spin*_ = 0.01, two-sided spin test) between these two modalities. **f**, Cell type enrichment of the weighted, ranked gene list from PLS analysis of covariation between MIND degree and gene expression, using the median loading rank within one of seven sets of genes, each characteristic of a canonical class of cells in the central nervous system: excitatory neurons (Neuro-Ex, *P* = 0.049), inhibitory neurons (Neuro-In, *P* = 0.17), endothelial cells (Endo, *P* = 0.81), astrocytes (Astro, *P* = 0.48), microglia (Micro, *P* = 0.02), oligodendrocytes (Oligo, *P* = 0.42) and oligodendroglial precursor cells (OPC, *P* = 0.30). The zero position on the *x* axis represents the median position of all 15,633 genes (position 7,816), with negative ranks indicating genes that have expression positively correlated with MIND node degree—that is, overexpressed at highly connected MIND network hubs. *P* values were FDR corrected after a two-sided permutation test controlling for both spatial autocorrelation in the brain MRI data and correlation structure in gene expression (*P < 0.05; see Methods for details).

MIND networks (parcellated by the DK template) demonstrated a remarkably strong correspondence with the brain transcriptomic co-expression network (Fig. 5). At the edge level, there was a greater than three-fold increase in correlations between edge weights of transcriptional similarity and MIND networks (Pearson’s *r* = 0.76, Spearman’s *ρ* = 0.81) compared to the equivalent correlations for MSNs (*r* = 0.23, *ρ* = 0.23). At the nodal level, there was an approximately two-fold increase in correlations between weighted degrees of transcriptional similarity and MIND networks (*r* = 0.85, *ρ* = 0.88) compared to the equivalent correlations for MSNs (*r* = 0.47, *ρ* = 0.30). A similar result was obtained when including the mean regional gray matter volume as a covariate (*r* = 0.75 for MIND networks, *r* = 0.5, for MSNs), suggesting that results were not driven by mean volume. We also observed an increased coupling between multivariate MIND networks and gene co-expression compared to both consensus DWI tractography from the HCP-YA cohort^39^ and univariate MIND networks based on CT only (Fig. 5d).

We tested the robustness of the strong relationships between MIND measures of structural similarity and transcriptional similarity through several sensitivity analyses: (1) constructing different transcriptional similarity networks based on all possible subsets of six donor brains (Fig. 5d and Supplementary Fig. 10); (2) changing the gene inclusion criteria based on varying thresholds of differential stability (Supplementary Fig. 10)^9^; and (3) replicating these analyses in the finer-grained DK-318 cortical parcellation (Supplementary Fig. 11). Under all conditions, we found that MIND network edge weights and weighted degrees remained strongly correlated with edge weights and weighted degrees of anatomically commensurate transcriptional similarity networks.

#### Cell-type-specific transcriptional profiles and MIND network degrees

To characterize the relationship between MIND degree and cell-typical gene expression, we used partial least squares (PLS) regression to relate the {15,633 × 34} matrix of regional gene expression with the {34 × 1} vector of group-averaged MIND network weighted degree. The first PLS component (PLS1) explained a significant amount of covariance (62% variance explained, *P*_*spin*_ = 0.01, using a ‘spin’ permutation test to correct for cortical spatial autocorrelation; Methods). Figure 5e shows the similarity between MIND degree and the cortical map of PLS-aligned transcription, calculated by averaging the spatial expression of all genes weighted by their PLS1 loadings.

Using published lists of genes specific to neuronal and glial cell types^10^, we calculated the median rank of genes in the PLS1 loadings within each cell-typical gene set, in line with prior enrichment work^10,11^. PLS1 was positively enriched for neuronal genes and negatively enriched for glial genes, with significant enrichment found for excitatory neurons and microglia (Fig. 5f). The result that MIND network hubs were located in cortical areas with high levels of neuron-typical transcription was consistent with the observation that MIND network degree was correlated with axonal connectivity in the macaque brain, given existing work demonstrating both higher tract-tracing connectivity between transcriptionally similar brain regions in mice^40^ and increased likelihood of connectivity between neurons with similar transcriptional profiles in *Caenorhabditis elegans*^41^.

### Heritability of structural similarity network phenotypes

To characterize the extent of genetic influences on structural similarity networks, we first estimated the twin-based heritability (${h}_{twin}^{2}$) for each of the five MRI features measured at each region and for each edge weight and weighted degree of the MSNs and MIND networks derived from them. Using 641 twin pairs (366 dizygotic and 275 monozygotic, total *n*_*twins*_ = 1,282) from the ABCD cohort, we fitted a standard ACE model to estimate additive genetic (A), shared environmental (C) and unique environmental (E) components of variance and to estimate twin heritability for each phenotype (Methods).

**Fig. 6:**
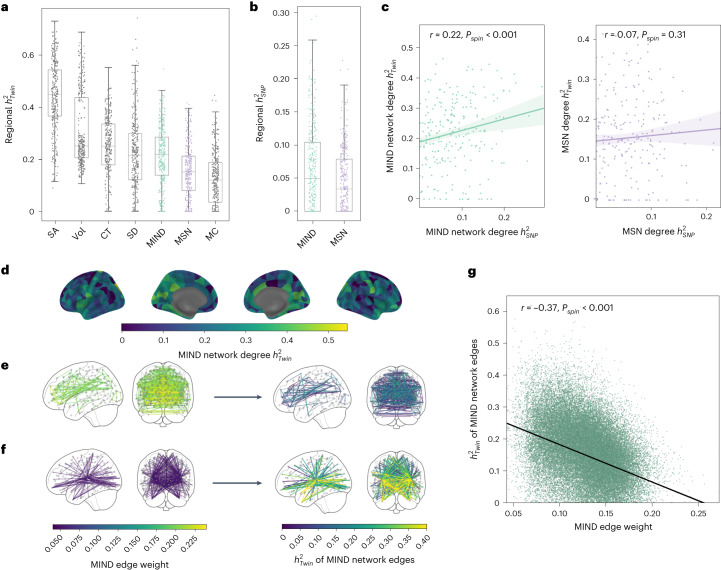
Estimating heritability, *h*^2^, of five regional MRI metrics and structural similarity network phenotypes derived from them. **a**, Twin-based heritability (${h}_{twin}^{2}$) of regional MRI metrics (SA, CT, Vol, MC and SD) and of weighted nodal degree for MIND networks and MSNs; each point represents one of 318 cortical areas. **b**, SNP-based heritability (${h}_{SNP}^{2}$) for weighted degree of MIND networks and MSNs (*n* = 318 regions). Box plots in **a** and **b** indicate data quartiles, and whiskers indicate the full data range, excluding outliers. **c**, Scatter plot of twin-based versus SNP-based *h*^2^ estimates for weighted degree of MIND networks and MSNs; each point represents a regional node in the cortical network. *P* values (uncorrected) were based on two-sided spin tests. Shading represents 95% confidence interval (CI) of best-fit lines. **d**, Cortical map of the regional ${h}_{twin}^{2}$ for MIND network degree. **e**,**f**, The strongest (**e**) and weakest (**f**) 1% of MIND edges and their corresponding ${h}_{twin}^{2}$ estimates. **g**, Scatter plot of ${h}_{twin}^{2}$ versus MIND network edge weights, with fitted line indicating significant negative correlation; each point is an edge in the network. The indicated *P* value was based on a two-sided spin test.

MIND demonstrated increased twin-based heritability compared to MSNs in terms of both edge weights (mean ${h}_{twin}^{2}=0.15$ versus 0.11, two-sided *t*-test, *P* < 0.001) and weighted nodal degree (mean ${h}_{twin}^{2}=0.21$ versus 0.15, two-sided *t*-test, *P* < 0.001) (Fig. 6a). To ensure that the higher heritability of MIND network phenotypes compared to MSNs was not due to differing relationships with brain size (see Supplementary Fig. 7 for details), we confirmed that MIND network degree demonstrated increased twin-based heritability compared to MSN degree (two-sided *t*-test, *P* < 0.001) after controlling for estimated total intracranial volume (eTIV).

The five regional MRI features had average twin-based heritabilities both higher and lower than the heritabilities of network phenotypes derived from them, ranging from ${h}_{twin}^{2}=0.44$ for SA to ${h}_{twin}^{2}=0.12$ for MC. The average heritability of MIND weighted degree (Fig. 6d) was significantly higher than ${h}_{twin}^{2}$ for regional MC (two-sided *t*-test, *P* < 0.001), similar to the ${h}_{twin}^{2}$ for regional estimates of mean SD (two-sided *t*-test, *P* > 0.05), and lower than the heritabilities of the three macrostructural MRI metrics related to the size of each regional node of cortex (SA, CT and Vol; two-sided *t*-tests, all *P* < 0.001). The cortical maps of regional MRI heritability for the different MRI features were positively correlated with each other (0.09 < *r* < 0.61; Supplementary Fig. 12). This result points to the existence of a general gradient of brain structural heritability, where similar anatomical patterns of heritability are observed across different MRI phenotypes.

#### Single-nucleotide polymorphism-based heritability

We estimated single-nucleotide polymorphism (SNP)-based heritability for weighted degree in MSN and MIND networks using genetic data from 4,085 unrelated individuals of predominantly European genetic ancestries from the ABCD cohort, and we used GCTA^42^ software for genome-wide complex trait analysis.

SNP-based heritability for weighted degree of MIND networks (mean ${h}_{SNP}^{2}=0.064$) was greater than for degree of MSNs (mean ${h}_{SNP}^{2}=0.046$), and this difference was significant (two-sided *t*-test, *P* < 0.001; Fig. 6b). SNP-based and twin-based heritabilities were positively correlated for weighted degree of MIND networks (*r* = 0.22, *P*_*spin*_ < 0.001) but were not correlated for degree of MSNs (*r* = 0.07, *P*_*spin*_ = 0.31) (Fig. 6c). This demonstrates that common genetic variants partly explain variance in MIND networks.

#### Increased heritability of MIND between dissimilar regions

Twin-based heritabilities for MIND network edges were robustly and negatively correlated with edge weights (*r* = −0.37; Fig. 6g). This is visualized in Fig. 6e,f, where the highest MIND edges, between the most similar areas of cortex—for example, inter-hemispheric connections—have much lower heritability than the lowest MIND edges, between the most dissimilar areas of cortex—for example, connections between neocortical areas and areas of insular and limbic cortex. We observed no correlation between Euclidean distance and edge heritability (*r* = 0.02, *P*_*spin*_ = 0.66), despite an exponentially decaying relationship between distance and MIND (Supplementary Fig. 3).

MIND network weighted degree was also negatively correlated with heritability (*r* = −0.24, *P*_*spin*_ = 0.02). When categorized by cytoarchitectonic class (Supplementary Fig. 12), weighted degree was more strongly heritable (mean ${h}_{twin}^{2}\ge 0.28$) for insular, primary sensory and limbic cortex and less strongly heritable for primary motor, association and secondary sensory cortex (mean ${h}_{twin}^{2}\le 0.22$). The difference in heritabilities between cytoarchitectonic classes was significant (ANOVA, *F*_6,311_ = 7.54; *P* < 0.001).

## Discussion

We present MIND network analysis as a method for distilling the large-scale, multidimensional, vertex-level data from structural brain MRI into a unified network model of cortical structure. These networks are technically reliable, map closely onto known principles governing cortical organization and can effectively detect individual differences in human connectomes due to both developmental changes and genetic variation.

At a methodological level, the relative superiority of individual brain connectome mapping by MIND networks compared to MSNs is simply explained. MIND measures similarity by the divergence between multidimensional distributions with many degrees of freedom, whereas MSNs are predicated on regional summary statistics of each MRI feature and are, therefore, less efficiently estimated with fewer degrees of freedom. Moreover, the regional *z*-scoring in MSN construction forces each feature to be equally variable across regions, which is biologically unrealistic, whereas MIND is driven only by structural features that truly differentiate cortical areas. These fundamental differences between MIND and MSN estimators of structural similarity greatly enhanced the reliability of the resulting MIND networks in terms of consistency between subjects, resilience to the inclusion of noise features and robustness to the choice of parcellation template used to define cortical nodes.

Benchmarking both MIND networks and MSNs against prior principles of cortical network organization^8,33,34^, we found that MIND networks were more representative of connections for left and right homologous regions, for regions belonging to the same cytoarchitectonic class and for regions with axonal interconnectivity demonstrated by the gold standard of retrograde tract tracing in the macaque monkey. These results consistently indicate that the connectomes rendered by MIND analysis of structural similarity are more aligned with the principles that structural similarity between regions should be greater for bilaterally homologous cortical areas, cytoarchitectonically homogenous areas and axonally connected areas. MIND networks were also more sensitive to age-related changes in structural architecture than either MSNs or diffusion tensor imaging (DTI) connectivity. This result suggests that the high between-subject consistency demonstrated for MIND networks does not preclude their sensitivity to detect developmentally relevant individual differences in cortical network organization.

Recent work has begun to establish another principle of brain network organization: that structurally similar or axonally interconnected regions will typically have more similar profiles of gene transcription than cytoarchitectonically dissimilar or unconnected pairs of regions^43^. In short, the structural architecture of the connectome recapitulates the organization of the brain gene co-expression network. We therefore expected—and confirmed—that the more reliable and valid connectomes produced by MIND analysis are more strongly correlated than MSNs or DTI networks with a gene co-expression network derived from the AHBA. Although the upper bound of the relationship between structural similarity and gene co-expression is unknown, the significantly greater strength of association between transcriptional similarity and structural similarity measured by MIND was evident at the level of both edges and nodes and across multiple parcellations. Moreover, the high-degree hubs of MIND networks were significantly co-located with areas where neuron-specific genes were highly expressed^10^.

These results strongly support the preferred use of MIND network analysis for future imaging studies designed to discover the transcriptional mechanisms underpinning anatomical connectomes in health and disease. However, several causal pathways could explain the strong coupling between MIND and transcriptional networks. Spatially patterned and developmentally phased gene expression drives the expansion and development of the human cortex^44^, so it is at least plausible that the network organization of transcription is an important driver or template of the network organization of the structural similarity and axonal connectivity of the cortex.

To investigate genetic effects on MIND phenotypes more directly, we demonstrated that MIND network edge weights and nodal degrees had higher twin-based and SNP-based heritabilities than similar MSN phenotypes. Notably, the heritability of the MIND similarity between two regions was found to be higher for edges between structurally dissimilar or differentiated regions—for example, edges connecting limbic, insular or primary sensory cortical areas to the rest of the network. Consequently, MIND network hubs in motor and association cortex, with a high degree of similarity to many other neocortical areas, were less genetically influenced than primary sensory, insular and limbic non-hub regions with more distinctive, less generally similar cytoarchitecture. These results are in line with previous work demonstrating increased heritability of brain structure in evolutionarily conserved, primary sensory regions as opposed to more recently developed heteromodal areas^45,46^. They are also complementary to recent work demonstrating that the heritability of functional network topography is stronger in unimodal as compared to association cortical regions^47^. Together, these results further endorse the biological validity of MIND networks compared to MSNs and set the scene for future, more detailed investigation of genetic effects on MIND network phenotypes.

One limitation of MIND networks—shared by MSNs—is that they do not currently include subcortical regions, which are not represented by surface reconstructions. In principle, however, MIND methodology could be extended to voxel-level metrics describing subcortical structures, such as microstructural metrics from DWI images. There are also current limitations in terms of our understanding of the optimal number and type of MRI metrics to measure for the purposes of MIND network analysis. In the present study, we mainly used five macrostructural MRI metrics that can be conveniently measured in widely available T1w MRI data from humans as well as the T1w/T2w ratio (a microstructural metric of intracortical myelination) in MIND analysis of macaque data. Although it is pragmatically encouraging to see that reliable and valid MIND networks can be estimated from standard T1w MRI data, it is plausible that MIND analysis based on a larger number and/or a more diverse range of MRI metrics might provide additional information about brain network organization.

Ultimately, we expect the study of MIND networks to provide a practical and informative new perspective on principles of cortical network organization that reflect the genetic architecture of the brain, with implications for mapping of individual human connectomes throughout normative and disordered processes of brain development and aging.

## Methods

### Ethics oversight

All data used for this study were previously published and collected in accordance with appropriate independent ethical approvals. Approval for our use of the ABCD data fell under a National Institute of Mental Health Data Archive (NDA) agreement, reflected in study 1796 on the NDA website.

### MIND estimation

#### Definition of the MIND similarity metric

Here we describe the definition of MIND as a statistical metric of structural similarity given a surface reconstruction of the cortex. This surface can be described by a set of vertices ${\bf{v}}_{i}\in {{{\mathcal{V}}}}$, where each ***v***_*i*_ is a vector of *d* structural features, such as CT and SD. These features (interchangeably described as structural and morphometric features) are automatically generated at the vertex level by FreeSurfer’s recon-all command. A cortical parcellation with *R* regions is a partition of ${{{\mathcal{V}}}}$ such that ${{{\mathcal{V}}}}=\{\{{{{{\mathcal{V}}}}}_{1}\},\ldots \{{{{{\mathcal{V}}}}}_{R}\}\}$. For each region *r*, we let *P*_*r*_ be the true multivariate distribution of structural features from which ${\bf{v}}_{i}\in {{{{\mathcal{V}}}}}_{r}$ are observations.

For a given pair of regions *a* and *b*, we estimate the KL divergence of *P*_*b*_ from *P*_*a*_, denoted as *D*_*KL*_(*P*_*a*_∥*P*_*b*_). Because KL divergence is not symmetric, we use a commonly used symmetric version of the metric, computed as follows and in line with previous work^14,51^:

1 $$D({P}_{a},{P}_{b})={D}_{KL}({P}_{a}\parallel {P}_{b})+{D}_{KL}({P}_{b}\parallel {P}_{a})$$

The value of *D*(*P*_*a*_, *P*_*b*_) corresponds to the value of *KL*(*a*,*b*) referred to in Fig. 1, which is used for simplicity in the diagram. We define the morphometric inverse divergence (MIND) metric of similarity, bounded between 0 and 1, as follows:

2 $$MIND(a,b)=\frac{1}{1+D({P}_{a},{P}_{b})}$$

#### Multivariate KL divergence estimation

One key challenge in calculating MIND networks is appropriately estimating multivariate KL divergence. Traditionally, KL divergence between empirical distributions is calculated by a two-step approach: (1) non-parametric estimation of the probability density functions (PDFs) of the observed data and (2) computation of the divergence using the approximated PDFs. However, the initial density estimation step of this approach is sensitive to many choices of parameters^14,15,18,51^. Extended to multiple dimensions, density estimation becomes especially problematic; for multivariate data with as few as three dimensions, standard non-parametric density estimators provide very poor results^52^. Although research into alternate methods for higher-dimensional density estimation is actively ongoing, no consensus currently exists on an effective, efficient method for this purpose^52^.

Here, we circumvent the need to perform the difficult first step of density estimation by leveraging a *k*-nearest neighbor approach^18^ for calculating multivariate KL divergence directly from the observed vertex-level data. This approach has considerable advantages compared to explicit density estimators—namely, it does not require the specification of any parameters, and it can be computed efficiently^53^.

More formally, given regions *a* and *b*, vertices *V*_*a*_ and *V*_*b*_, with true multivariate distributions *P*_*a*_ and *P*_*b*_, the KL divergence between *P*_*a*_ and *P*_*b*_ is defined mathematically as follows:

3 $${D}_{KL}({P}_{a}\parallel {P}_{b})={\int}_{{{\mathbb{R}}}^{d}}{p}_{a}({{{\bf{x}}}})\log \frac{{p}_{a}({{{\bf{x}}}})}{{p}_{b}({{{\bf{x}}}})}d{{{\bf{x}}}}\ge 0$$

We used the *k*-nearest neighbor divergence approximation by Perez-Cruz^18^ to estimate *D*_*KL*_(*P*_*a*_∥*P*_*b*_):

4 $${\widehat{D}}_{KL}({P}_{a}\parallel {P}_{b})=-\frac{d}{n}\mathop{\sum }\limits_{i=1}^{n}\log \frac{{r}_{k}\left({{{{\bf{x}}}}}_{i}\right)}{{s}_{k}\left({{{{\bf{x}}}}}_{i}\right)}+\log \frac{m}{n-1}$$

Here, *d* is the number of structural features used, $n=\parallel {{{{\mathcal{V}}}}}_{a}\parallel$ and $m=\parallel {{{{\mathcal{V}}}}}_{b}\parallel$, and ${r}_{k}\left({{{{\bf{x}}}}}_{i}\right)$ and ${s}_{k}\left({{{{\bf{x}}}}}_{i}\right)$ are the Euclidean distances of **x**_*i*_ to the *k*-th most similar vertex of **x**_*i*_ in ${{{{\mathcal{V}}}}}_{a}\setminus {{{{\bf{x}}}}}_{i}$ and ${{{{\mathcal{V}}}}}_{b}$, respectively (where ${{{{\mathcal{V}}}}}_{a}\setminus {{{{\bf{x}}}}}_{i}$ is the set ${{{{\mathcal{V}}}}}_{a}$ with the sample **x**_*i*_ removed). We use *k* = 1 (nearest neighbor) in our analysis, and we calculate ${r}_{k}\left({{{{\bf{x}}}}}_{i}\right)$ and ${s}_{k}\left({{{{\bf{x}}}}}_{i}\right)$ efficiently using *k*-dimensional trees, a method for data representation that enables the rapid lookup of nearest neighbors.

To account for the unlikely but possible occurrence that the estimation of KL is negative, we set the minimum value of the estimate to be 0. A symmetric measure of KL divergence was then given by:

5 $$\widehat{D}({P}_{a},{P}_{b})=\max ({\widehat{D}}_{KL}({P}_{a}\parallel {P}_{b}),0)+\max ({\widehat{D}}_{KL}({P}_{b}\parallel {P}_{a}),0)$$

And MIND was finally estimated by:

6 $$MIND(a,b)=\frac{1}{1+\widehat{D}({P}_{a},{P}_{b})}$$

#### Standardizing and filtering vertex-level data

Because each feature is measured on different scales, we standardized (*z*-score) each feature across all vertices in the brain before parcellating the data into vertex-level distributions and calculating MIND.

Additionally, structural vertex-level data can sometimes represent biologically unfeasible conditions—namely, when vertices have values of 0 for CT, Vol or SA. For MIND estimation, we discarded all such vertices. One result of this filtering step is that if a region is left with zero or one vertices, a complete MIND network cannot be computed. Thus, using parcellations of smaller parcel size will generally lead to higher likelihood that one or more regions contains no vertices and, therefore, fewer networks that can be fully calculated. Removing the condition that all vertices must have above-zero values of thickness, volume and area will mitigate this, although at the tradeoff of including vertices that correspond to potentially unfeasible conditions.

#### Computational costs of MIND network analysis

Construction of MIND networks can be completed at reasonable computational cost. Given *d* dimensions and *n* vertices, a *k*-dimensional tree can be constructed in ${{{\mathcal{O}}}}(dn\log n)$—with more recent approaches further improving a worst-case construction time to ${{{\mathcal{O}}}}(n(d+\log n))$ (ref. ^53^)—and can be queried in ${{{\mathcal{O}}}}(\log n)$ (refs. ^54,55^). This computation is not a bottleneck; in practice, we observed that the computational resources expended during structural image (FreeSurfer) pre-processing far exceeded MIND network computation. For reference, on consumer hardware, computation of a single MIND network ranged from roughly 1 min (68-region of interest (ROI) DK atlas) to roughly 10 min (360-ROI HCP atlas).

#### MSN calculation

MSNs were computed as described in Seidlitz et al.^6^. Specifically, we considered the widely used summary statistics computed by FreeSurfer’s mris_anatomical_stats command to characterize each region. The five summary statistics describing each region are the following:

- Mean sulcal depth
- Mean cortical thickness
- Total volume
- Total surface area
- Integrated rectified mean curvature

Each feature was *z*-scored, and the resulting MSN was defined as the pairwise Pearson correlation between all vectors of the five standardized features. To construct MSNs with ${{{\mathcal{N}}}}(0,1)$ noise features (as in Fig. 2f), the Gaussian noise columns were added as new features at each vertex, averaged within each region and *z*-scored across regions before inclusion into the vector of structural features.

### Human MRI datasets

#### ABCD imaging dataset

The ABCD cohort currently comprises T1w structural MRI data on 11,449 participants (including 697 twin pairs) ages 9–11 years at baseline scanning. Recruitment for the ABCD study was intended to generate a diverse, representative sample^56^ for the longitudinal study of brain development and cognition. This work is registered as study 1796 on the NDA (10.15154/1528079). The number of subjects included for different analyses can be visualized in Supplementary Fig. 2. After data filtering and quality control (QC), this led to 10,367 subjects included in our principal dataset parcellated in DK-318 (*n* = 10,353 for DK and *n* = 9,218 for HCP parcellation, with the difference between parcellation due to the vertex filtering step described above). Group-level MIND networks and MSNs were constructed from these cohorts. These 10,367 subjects in DK-318 parcellation included 641 complete twin pairs (366 dizygotic and 275 monozygotic), which served as the cohort for estimating twin-based heritability. To estimate SNP-based heritability, we used a sample of 4,085 subjects, comprising unrelated participants of European ancestry with MRI and genetic data that passed QC criteria. To study the effect of including varying numbers of noise columns (Fig. 2f), we used a random subset of 150 subjects to avoid the cost of constructing many network versions for all 10,367 individuals.

Extensive documentation of the scanner types and protocols used for MRI in the ABCD study can be found in Hagler et al.^19,57^. T1w images were 1-mm isotropic, radiofrequency (RF)-spoiled gradient echo using prospective motion correction if available and from one of three (3T) scanner models: Siemens (Prisma VE11B-C), Philips (Achieva dStream, Ingenia) or GE (MR750, DV25-26)^19,48^. The images were processed using FreeSurfer version 5.3.0.

#### ABCD MRI QC

To ensure high quality of the included scans, we used the Euler number^1^, an index of scan quality generated automatically by FreeSurfer. Supplementary Fig. 2 shows the distribution of Euler number in the entire ABCD cohort, with some extreme outliers in the sample. To discard these scans, we used a cutoff threshold of −120, corresponding to a median absolute deviation (MAD) score of ≥2.6.

#### ABCD site-related batch effects

ABCD is a multi-site study with well-known batch effects due to scanning at different sites^58^. To correct these site-related batch effects in quality controlled data, before MIND analysis, we used neuroCombat version 0.2.12 (ref. ^59^), an adaptation of the standard ComBat batch correction tool^60^ designed specifically for structural MRI brain data. While adjusting for site-specific effects in this manner, we included age (in months) and sex to be biologically relevant covariates (that is, differences in age and sex distribution between sites were not considered site-specific effects).

#### HCP-YA and HCP-D imaging datasets

The HCP-D cohort aims to provide MRI scans for over 1,300 participants ages 5–21 years to shed light on brain development over adolescence^21^. The data used in this work were part of release 1.0, containing cross-sectional images (pre-processed using FreeSurfer version 6.0) from 655 subjects ages 8–21 years (49% male). All 3T images were acquired on a Siemens Prisma scanner 80-mT/m gradient coil, multiecho, and with 0.8-mm isotropic resolution. Full imaging acquisition parameters are described in detail in Harms et al.^61^ and Somerville et al.^21^. Pre-processed DWI images were not yet available at the time this work took place and, hence, were not considered.

We used data from the HCP-1200 release of the HCP-YA cohort. This release provides cross-sectional 3T images (pre-processed using FreeSurfer version 5.3.0-HCP as described in Glasser et al.^62^) from 1,113 young adults ages 21–35 years. The image acquisition parameters are described in detail elsewhere^20,62^; in short, images were 0.7-mm isotropic, field of view (FOV) 224 × 224 mm, TI = 1,000 ms and TR = 2,400 ms. Connectivity matrices based on diffusion tractography were published by Arnatkevičiūtė et al.^24^. Detailed pre-processing steps are provided in the original publication; in summary, processing of DWI images was performed by Arnatkevičiūtė et al.^24^ using MRtrix3 (ref. ^63^), FSL with FMRIB Software Library^64^, iFOD2 (ref. ^65^) and anatomically constrained tractography (ACT). Connectivity strengths were based on the mean fractional anisotropy within the voxels of streamlines between cortical areas. DTI connectivity images were provided in the HCP 360-region parcellation and were available for only a subset of the entire cohort, so our final total number of subjects with all imaging modalities was *n* = 960 (46% male).

To replicate age prediction results from Fig. 4 on DTI networks processed through a separate processing pipeline, we additionally used the publicly available individual HCP-YA DTI dataset processed by Rosen et al.^25^, also in the HCP 360-region parcellation. Processing for these data used a separate method of probabilistic tractography estimation and estimated structural connectivity using streamline counts. In line with the original publication, we considered structural connectivity to be the log-transformed probabilistic tractography values, adding jitter of 1 × 10^−10^ to ensure that all entries are defined after log transformation. Rosen et al.^25^ provided a more detailed discussion of processing steps in the original publication.

To compare consensus DTI connectivity with the network of gene co-expression, a population-averaged DTI connectome was needed in the DK parcellation (not provided by Arnatkevičiūtė et al.^24^ or Rosen et al.^25^). We therefore leveraged the population-level DTI connectome in DK parcellation pre-processed and provided by the ENIGMA consortium as part of the ENIGMA toolbox^39^. The original publication details all pre-processing steps, including using MRtrix3 with ACT and spherical deconvolution informed filtering of tractograms (SIFT2)^66^. Structural connectivity was estimated using fiber density as the measure of structural connectivity.

#### Cortical parcellations

We used the DK^22^, DK-318 (ref. ^23^) and HCP^31^ parcellations in this work. Unlike the DK and DK-318 parcellations, which are based entirely on anatomical landmarks from structural MRI, the HCP-Glasser parcellation is in part defined by functional connectivity estimated from functional MRI (fMRI) data. As such, it may transgress structurally defined cortical areas, so conclusions drawn about node-specific properties should ensure generalizability to alternative parcellations defined using structural landmarks alone. To compare edge-wise consistency between group-level DK and DK-318 atlases (Fig. 2h), we assumed that an edge between two regions in the DK atlas should be similar to that derived by averaging all edges between the subdivisions of the regions in the finer-grained DK-318 atlas. In this manner, we interpolated the group DK-318 networks back to the original DK atlas and then compared these recreated networks to those computed directly on the original 68-region DK parcellation. The correlation between the original DK and interpolated (DK-318 interp.) networks was used to measure edge-level parcellation consistency.

The mapping from each region in the 318-region subdivision of the DK atlas (Fig. 2i) was based on the mapping used in Seidlitz et al.^6^, originally performed by Vértes et al.^67^ and Whitaker et al.^68^. These prior studies used the closely related DK-308 parcellation^23^, which is an asymmetric version of the DK-318 atlas. We used a simple majority-voting procedure to translate the DK-308 parcellation to the related DK-318 atlas. Comparisons to individual DTI connectomes were performed using the HCP-360 due to the availability of publicly available, externally processed data.

#### Age prediction

We trained machine learning models to predict the age of participants in either the HCP-D or HCP-YA cohorts using node degree or edge weights of MSNs, MIND networks and DTI connectivity matrices. To align with most of the other analyses in the paper, the DK-318 parcellation was used for the HCP-D cohort, where DTI was not available. For the HCP-YA cohort, we used the HCP 360-region parcellation to match the parcellation scheme provided by Arnatkevičiūtė et al.^24^ and Rosen et al.^25^. All models were trained on 10 train/test splits (90% train data, 10% test data) with non-overlapping test sets. Models were implemented in Python 3.6 using the sklearn package (version 0.24.1)^69^. Models trained on node degree used five-fold cross-validation for each training set over a set of nonlinear and linear models—specifically, a support vector machine with an RBF kernel (sklearn specification: SVR(kernel = ‘rbf’)) and C regularization values of 0.1, 1.0, 10 or 100 and a linear Gaussian process (GP) regression model with a summed linear and noise kernel (sklearn specification: GaussianProcessRegressor(kernel=DotProduct() + WhiteKernel(noise_level_bounds=(1e-10, np.inf)). The linear GP is equivalent to a Bayesian linear regression, with the noise kernel modeling the presence of i.i.d noise. All training sets were standardized using the StandardScaler() function from sklearn; test sets were accordingly transformed using the normalization function estimated on the training set. For models trained on all individual edges, due to the very large number of features (>50,000 features), we used the GP regression model alone, as in Morgan et al.^70^.

To evaluate model performance while ensuring that predictions were not biased by the presence of confounds related to subject age, we used the partial Spearman correlation of predicted versus true age, controlling for the effect of sex, Euler number (a measure of scan quality that is known to have a strong relationship with age^25^) and a global matrix coefficient (defined as the sum over the entire connectivity matrix, which may detect global measures such as total intracranial volume, as discussed in Supplementary Fig. 7). This post hoc adjustment ensured proper correction for the potential effect of confounds by avoiding the statistical issues that arise when regressing confounds from feature space before model training^71^.

### Twin-based heritability

We used the umx package (version 2.10.0) using R (version 4.1.3) to implement a structural equation model of the ACE model to infer heritability estimates^72,73^. The ACE model estimates the contributions to observed variance due to additive genetic (A) in the context of common (C) and specific environmental (E) effects on variance^72,73^. We defined heritability (*h*^2^) as the proportion of variance due to additive genetics contributions, such that ${h}^{2}=\frac{A}{A+C+E}$ (ref. ^74^).

The ACE model does not estimate A, C and E directly but, rather, estimates path coefficients *a*, *c* and *e*, such that *a*^2^ = A, *c*^2^ = C and *e*^2^ = E. These path coefficients are sometimes directly used as reports of heritability (for example, Bethlehem et al.^2^). To validate our processes for twin-based heritability estimation, we replicated a previously reported estimate of ${h}_{twin}^{2}=0.58$ for whole-brain gray matter volume (GMV) in the ABCD cohort^2^. The published path coefficients corresponding to this heritability estimate were the following: *a* = 0.745, *c* = 0.624 and *e* = 0.238. We calculated values of *a* = 0.765, *c* = 0.596 and *e* = 0.246, which were highly consistent.

### Human genetic data QC and SNP-based heritability

Full details of genetic QC procedures are provided by Warrier et al.^75,76^. In brief, we excluded SNPs with genotyping rate <90% and individuals with genotyping rate <95%, whose genetic sex did not match their reported sex. We identified individuals of predominantly European genetic ancestries using multidimensional scaling after including samples from the 1000 Genomes Project phase 3 data^77^. In the subset of individuals of predominantly European ancestries, we further excluded SNPs not in Hardy–Weinberg equilibrium (*P* < 1 × 10^−6^) and individuals with excessive heterozygosity. Related individuals (>5% identity by state) were excluded using Genome-wide Complex Trait Analysis Genome-based Restricted Maximum Likelihood (GCTA-GREML) software (version 1.93)^42^ before estimation of SNP-based heritabilities using a genetic relatedness matrix. The genetic relatedness matrix was derived from genotyped samples after controlling for age, age^2^, age × sex, age^2^ × sex, sex, imaging center, mean framewise displacement, maximum framewise displacement, Euler Index and the first 10 genetic principal components as covariates.

### Macaque MRI and tract-tracing data

We used MRI data from 19 female rhesus macaque monkeys (*Macaca mulatta*, ages 18.5–22.5 years) in the UC-Davis cohort provided by the PRIME-DE resource^30^. The animals were anesthetized and scanned on a Siemens Skyra 3T MRI with a 4-channel clamshell coil with 0.3 isotropic resolution (TR = 2,500 ms)^30^. These data were pre-processed using the HCP Non-Human Primate^78^ pipeline by Xu et al.^29^.

All individual scans were previously spatially co-registered with the group-level Yerkes19 atlas^79^. On this basis, we constructed group-level structural similarity networks by first averaging vertex-level features and then constructing a MIND network and an MSN. We calculated MSNs by manually generating the same output as performed by FreeSurfer’s mris_anatomical_stats command used to calculate human MSNs. Specifically, within each region, vertex values of SD and CT were averaged; Vol and SA were summed; and MC was absolute-valued, multiplied by SA and summed (thus outputting integrated rectified MC).

We used four tract-tracing connectivity matrices based on the 91-region Markov M132 parcellation of the left hemisphere^27^: the original and most widely used {29 × 29} complete connectivity matrix between 29 cortical areas used both as source and target regions in retrograde tract-tracing experiments; the {29 × 91} matrix including all originally measured source–target connections^27^; and the corresponding {40 × 40} and {40 × 91} matrices from a recently published extension of the original Markov dataset, which increased the number of target regions from 29 to 40 (ref. ^28^). For all these tract-tracing connectomes, we used the log-transformed fraction of labeled neurons (log(FLNe)) as the measure of axonal connectivity^6,27^. Additionally, we used a bi-hemispheric connectivity matrix based on the independent regional mapping (RM) parcellation and estimated by using DWI to infer the connectivity weights from categorical estimates derived from the CoCoMac database^50,80^.

To study the relationship between structural similarity and regional connectivity profiles, we generated two tract-tracing connectivity profiles per region based on the vectors of afferent and efferent edges connected to a node. We correlated both of these vectors with the node’s (undirected) profile of structural similarity (MIND or MSN), Fisher transformed the two correlations and averaged and inverse transformed to calculate a final correlation between a region’s tract-tracing connectivity and structural similarity (reported in Fig. 3c).

To test for the difference between the correlation between MIND networks and tract tracing versus that between MSNs and tract tracing, we performed edgewise bootstrapping with the one-sided null hypothesis that MIND did not have a greater correlation than MSNs with tract tracing. Thus, for each bootstrapped edge sample, we calculated the difference between the tract-tracing correlation for MIND and MSNs and then calculated the *P* value as the fraction of samples for which this value fell below 0. We used a significance threshold of *α* = 0.01 corresponding to a Bonferroni correction for the five connectomes used.

### Gene expression analysis

#### Data and pre-processing

The AHBA contains high-resolution spatial genome transcriptional data in the cortex from six postmortem brains (male/female = 4/2, mean age = 45 years). We focused our analysis of the AHBA on the DK parcellation of human brain gene expression maps, given prior work on standardizing the pre-processing pipeline for this atlas (Arnatkevičiūtė et al.^9,38,81^) and because the coarse-grained DK parcellation ensures high donor coverage for all regions. Only two brains provided data from the right hemisphere, so we focused on the left hemisphere only, and we used the abagen package (version 0.1.3) developed by Markello et al.^81^ with default settings to fetch (the get_expression_data command) and manipulate the AHBA data. These pre-processing steps included aggregating probes across all available donors, selecting probes using an intensity-based filtering threshold of 0.5, normalizing microarray expression values for each sample and donor using the scaled robust sigmoid function and combining gene probes for each region within each donor before combining across donors.

#### Transcriptional similarity metric

We used an angular similarity metric based on cosine distance (rather than raw cosine similarity or Pearson correlation) to measure transcriptional similarity between regions. If **g**_*x*_ and **g**_*y*_ are the vectors of gene expression for regions *X* and *Y*, the transcriptional similarity, *τ*, between the two regions is defined as:

7 $$\tau ({\bf{g}}_{x},{\bf{g}}_{y})=\left(1-\arccos \left(\frac{{\bf{g}}_{x}\cdot {\bf{g}}_{y}}{\parallel {\bf{g}}_{x}\parallel \parallel {\bf{g}}_{y}\parallel }\right)/\pi \right)$$

The choice of this metric was informed by the previous finding^82^ that it more precisely measures differences between high-dimensional vectors with high average similarity, which is the case for regional transcription data. In this formulation, the ∥ ⋅ ∥ operator represents the length of the vector (the square root of the sum of squares of all elements). In the case of no gene filtration, all 15,633 genes are used for each region.

#### Cell-type-specific gene enrichment analysis

To perform enrichment analysis of the genes most highly co-located with MIND degree, we first performed a PLS regression between the {15,633 × 34} matrix of AHBA gene expression and the {34 × 1} vector of MIND weighted node degree and then ranked each gene based on their position in the list of PLS1 loadings, with lower rank corresponding to genes with higher positive correlation with MIND degree. We then leveraged the extensive meta-analysis performed by Seidlitz et al.^10^ to assign 4,110 genes to one of seven major classes of cells in the central nervous system: excitatory neurons, inhibitory neurons, endothelial cells, astrocytes, microglia, oligodendrocytes and oligodendroglial precursor cells. To measure the cell type enrichment in the loadings of the first component of the PLS, we calculated the median rank of each set of cell-typical genes (as in refs. ^10,11,83^). Then, we used a permutation test to account for both the intrinsic correlation structure in the AHBA expression data as well as spatial autocorrelation in the cortical map of MIND degree, as detailed below.

### Spin permutation tests

We adopted the widely used ‘spin’ test to measure for significance of association between two cortical maps while correcting for spatial autocorrelation^84,85^. This test uses the (*x*,*y*,*z*) coordinates of each parcel to generate permutations of parcellated data that maintains its spatial embedding. We used the implementation by the gen_spinsamples command from the netneurotools Python package with the parameter method set to hungarian, which ensures that each index is used only once per permutation and uses the Hungarian algorithm to minimize the global cost of reassignment. The same spatial permutations were applied to the left and right hemispheres to maintain bilateral symmetry. When testing for significance between network edges (that is, the relationship between MIND edge heritability and mean edge strength), we used the same permutation scheme and simply applied spatial permutations to both node sources and targets (that is, the rows and columns of a connectivity matrix), thus, in effect, rotating the entire network. All such statistical tests used were two-sided, such that the null hypothesis was that the variance explained (*r*^2^) between two cortical maps or edges was not greater than the *r*^2^ expected by chance, accounting for spatial autocorrelation. We used 1,000 permutations for all tests.

To test for the significance of cell-typical gene set enrichment in the loadings from the PLS component between gene expression and MIND degree, we generated 1,000 spin test permutations of the vector of MIND degrees while keeping the gene expression data intact. For each spatial permutation of MIND degree, we fit a new PLS model and ensured that lower loading rank corresponded with a positive correlation with the permuted brain map. For each new model, we calculated the median gene rank within each set of cell-type-specific genes. The two-sided null hypothesis of our permutation test was that the median gene rank of a cell-typical gene set was not significantly different from the median position of all genes (rank 7,816). We, thus, calculated the *P* value as the fraction of all permutations for which each set of cell-typical genes had a median rank farther away from rank 7,816 than the true median rank. We false discovery rate (FDR) corrected the resulting seven *P* values. In this scheme, gene sets with median PLS rank significantly lower than the median position were positively associated with MIND degree.

### Statistics and reproducibility

To ensure reproducibility of our results, we replicated our central findings from the ABCD (Fig. 2) in the HCP-YA dataset (Supplementary Fig. 8), replicated our age prediction results from the HCP-D dataset in the HCP-YA dataset (Fig. 4) and replicated the performance of age prediction using DTI connectomes in HCP-YA using data processed through two separate pipelines (Supplementary Fig. 9). No statistical method was used to predetermine sample size in our analyses, but our sample sizes are similar to those reported in previous publications^19–21^. Subject exclusion criteria are described in the dataset descriptions (Methods) and Supplementary Fig. 2. Data distributions in Figs. 4b,c and 6a,b and Supplementary Fig. 9 were assumed to be normal, but this was not formally tested.

### Reporting summary

Further information on research design is available in the Nature Portfolio Reporting Summary linked to this article.

## Online content

Any methods, additional references, Nature Portfolio reporting summaries, source data, extended data, supplementary information, acknowledgements, peer review information; details of author contributions and competing interests; and statements of data and code availability are available at 10.1038/s41593-023-01376-7.

## Supplementary information

Supplementary Information Supplementary Table 1 and Supplementary Figs. 1–14.

Reporting Summary

## Data Availability

The pre-processed macaque data^29,30^ can be accessed at https://balsa.wustl.edu/reference/976nz. Tract-tracing connectomes based on the Markov parcellation can be accessed at https://core-nets.org. The multimodal connectome using the RM parcellation (as well as the RM atlas itself) can be accessed at https://zenodo.org/record/1471588#.YqBt5S2ca_U. Data from the ABCD cohort require access to the NIMH Data Archive and can be applied for at https://nda.nih.gov/abcd. HCP-YA data can be accessed and downloaded at https://www.humanconnectome.org. Individual DTI connectomes provided by Arnatkevičiūtė et al.^24^ for the HCP-YA dataset can be downloaded at https://zenodo.org/record/4733297#.Y8wVoS-l368. HCP-YA connectomes used for replication, processed by Rosen et al.^25^, can be accessed at https://zenodo.org/record/4060485#.Y858GS-l0Q0. HCP-D data can be accessed and downloaded by following the instructions at https://www.humanconnectome.org/study/hcp-lifespan-development/data-releases. Consensus HCP-YA DTI connectivity in DK parcellation can be downloaded directly from the ENIGMA toolbox at https://enigma-toolbox.readthedocs.io/en/latest/. Expression data from the Allen Human Brain Atlas can be downloaded using the abagen package^81^ at https://abagen.readthedocs.io/en/stable/.

## Code availability

Python code for MIND calculation (using subjects processed through FreeSurfer) is publicly available at https://github.com/isebenius/MIND and 10.5281/zenodo.7974716. The supplied code supports the use of standard surface-level features as well as custom features in surface format. The repository includes additional functionality to include volumetric features into MIND computation.

Code used for downstream data analysis was performed using Python version 3.6 using publicly available packages, including sklearn (0.24.1), numpy (1.17.1), scipy (1.5.4) and pandas (0.25.1)^69,86–88^.
