# Supplementary material for: Robust estimation of cortical similarity networks from brain MRI
Source: Nat Neurosci. 2023 Jul 17;26(8):1461–71. doi: 10.1038/s41593-023-01376-7 (PMC10400419; doi:10.1038/s41593-023-01376-7)
Supplement: Supplementary file 1 — Supplementary Table 1 and Supplementary Figs. 1–14. [file 41593_2023_1376_MOESM1_ESM.pdf]

nature neuroscience

Technical Report https://doi.org/10.1038/s41593-023-01376-7

Robust estimation of cortical similarity networks from brain MRI

In the format provided by the authors and unedited

Supplementary Materials

Inventory

In this supplement, we provide additional information to illustrate our workflow, more comprehensively characterize MIND networks and MSNs, and report sensitivity analyses and other results aimed at contextualizing the main text as follows:

- Figures S1 and S2: additional figures to clearly illustrate the pipeline for constructing a MIND network from a raw T1w image, and to visualize our subject inclusion criteria at each stage of analysis.
- Figures S3, S4, S5, S6, S7: additional analyses and figures characterizing MIND networks and MSNs, including their connection to Euclidean distance, their association with brain size, and their dependence on specific structural features.
- Figures S8, S9, Table S1: replication of several validation steps in the HCP-YA cohort and comparison to other connectivity methods, alongside additional results and reporting concerning age prediction in the HCP-YA and HCP-D cohorts.
- Figures S10 and S11: additional sensitivity analyses concerning the coupling between networks of transcriptional similarity from the Allen Human Brain Atlas (AHBA) and both MIND networks and MSNs.
- Figure S12: additional results to describe how the patterning of MIND heritability corresponds to neuroanatomical features and MIND network phenotypes.
- Figure S13: additional results demonstrating the added benefit of a multivariate MIND approach over univariate vertex-level approaches in terms of correspondence to macaque tract-tracing connectivity.
- Figure S14: a figure comparing the group-level MIND network and MSN from the HCP-YA and ABCD cohorts.

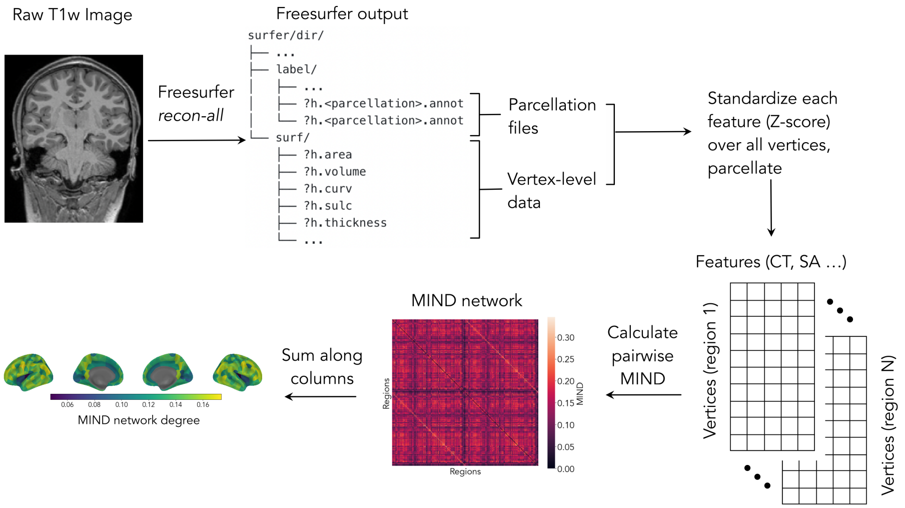

Fig. S1 A visualization of the entire MIND pipeline, from raw T1w image to MIND network phenotypes, using FreeSurfer as the image-processing tool of choice. Using the T1w nifti file as input, we processed the scan using FreeSurfer's **`recon-all`** command. This outputted **`label/`** and **`surf/`**, the folders in the surfer directory containing all necessary information for MIND calculation. The **`surf/`** folder housed all vertex-level data, with **`?h.area`**, **`?h.volume`**, **`?h.curv`**, **`?h.sulc`**, and **`?h.thickness`** containing the vertex-level values of surface area, grey matter volume, mean curvature, sulcal depth, and cortical thickness, respectively. Additional, custom surface-level features could also be used in MIND calculation as described in the code documentation. The **`label/`** folder housed the **`?h.annot`** files necessary to parcellate the vertex-level data. If the use of a non-standard parcellation was required, additional **`?h.annot`** files could be created using e.g. the `mris_ca_label` command. Given these outputs, we standardized (Z-score) each feature across all vertices, then parcellated the vertex data to create data-frames of vertices of all features per region. Calculating the MIND similarity statistic then produces the final MIND network, and the weighted nodal degree was then computed as the sum or average along the columns.

	HCP-YA		HCP-D	
Method	Degree	Edge	Degree	Edge
DTI	0.05 (0.07)	0.20 (0.07)	N/A	N/A
DTI-rep	0.11 (0.10)	0.17 (0.09)	N/A	N/A
MSN	0.10 (0.11)	0.26 (0.09)	0.33 (0.07)	0.67 (0.08)
MIND	**0.23** (0.09)	**0.31** (0.06)	**0.65** (0.07)	**0.76** (0.06)

Table S1 Prediction performances for all models across both HCP-YA and HCP-D datasets. Mean test performances (partial Spearman correlation) are reported, with standard deviation in parentheses. "DTI" refers to the DTI provided by Arnatkevičiūtė et al. (2020), whereas "DTI-rep" refers to the data provided by Rosen et al. (2017).

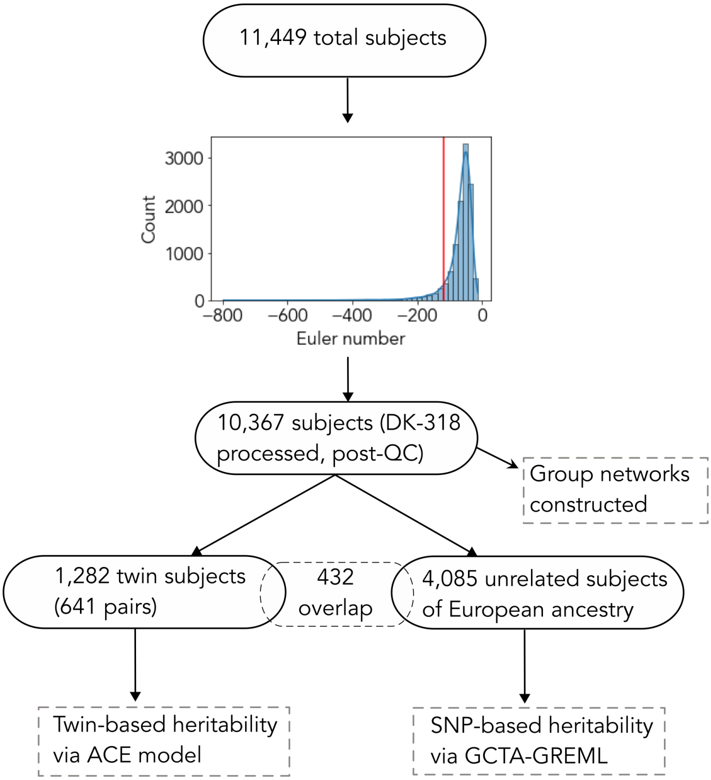

Fig. S2 Subjects included for each analysis. Flow chart indicating the total number of subjects included for construction of the group-level networks, and for both heritability analyses. The histogram shows the Euler number QC cutoff threshold of -120, corresponding to a median absolute deviation of 2.6. The 10,367 post-QC subjects listed were those who had a) an Euler number over -120 and b) no regions assigned zero vertices in the DK-318 parcellation. For the twin-based heritability analyses, any triplets were excluded.

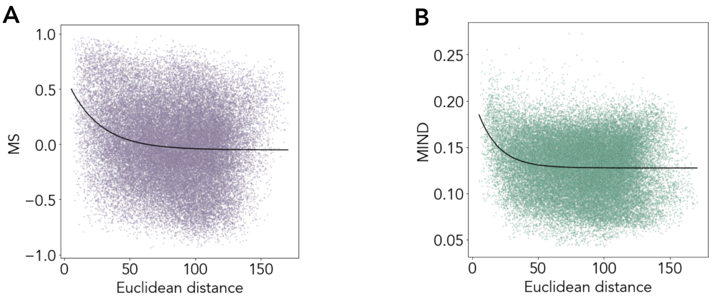

Fig. S3 Relationship to Euclidean distance. The exponentially decaying relationship between Euclidean distance and edge strength for group-level morphometric similarity networks (A) and MIND networks (B), DK-318 parcellation.

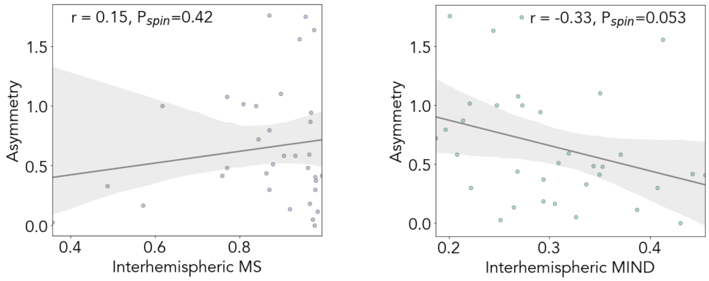

Fig. S4 Interhemispheric structural similarity and asymmetry of cortical surface area. Scatterplots indicating the relationship between published values of asymmetry between the surface area of homologous regions of the left and right hemispheres and either group mean interhemispheric morphometric similarity (left panel) or MIND (right panel) estimated between homologous regions. P-values were uncorrected and based on two-sided spin tests. Shading represents 95% CI of a line of best fit. The y-axis indicates the magnitude of asymmetry, with higher values corresponding to regions with larger overall asymmetry. The map of surface area asymmetry was based on an analysis of 17,141 subjects by Kong et al. (2018). These published brain maps (in the DK parcellation) assigned a Z-score to each region, indicating the magnitude and direction (left or right bias) in surface area. We defined regional asymmetry as the absolute value of these published directed Z-scores. MIND networks demonstrate the theoretically expected relationship - with higher levels of structural similarity between more symmetric cortical areas - more clearly than MSNs.

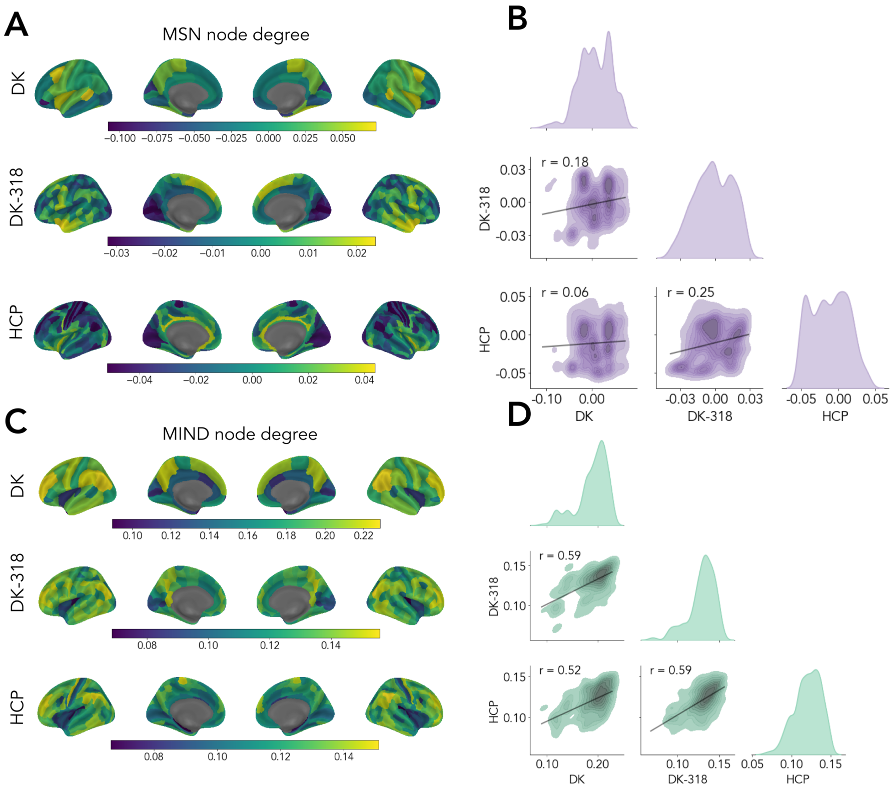

Fig. S5 Robustness to changes in parcellation. A, C) The weighted nodal degree of MIND networks and MSNs for Desikan Killiany (DK), DK-318, and HCP parcellations. B, D) Associations between weighted degree distributions for all pairs of DK, DK-318, and HCP parcellations. The correlations were calculated by mapping from regional to vertex space as described in Figure 2. These plots present a more detailed visualization of the same values reported in Figure 2F. Diagonal entries show the degree distribution for each network type and parcellation.

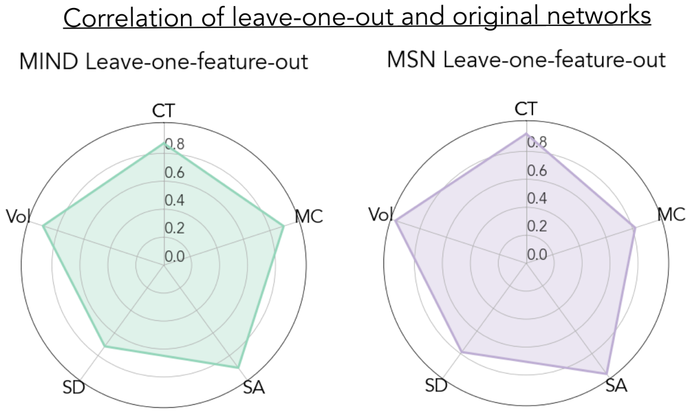

Fig. S6 Sensitivity of MIND networks and MSNs to exclusion of individual MRI features. Radar plots demonstrating the effect on overall network structure of leaving each specific MRI feature out of the estimation of structural similarity. For both MIND networks and MSNs, we constructed group-averaged MIND networks and MSNs using a leave-one-out scheme. Each radial position indicates the feature left out of the network construction. The correlation between the resulting four-feature network and the original five-feature network is indicated by the radial length. Replicating the analogous original findings from Seidlitz et al. (2018), we found that the consistency of leave-one-out with group-network architecture was fairly high for all feature subsets, and similar for both MIND networks and MSNs. For both methods, sulcal depth was the feature that most strongly influenced group-average network structure (edgewise $r = 0.71$ vs. 0.79 for MIND networks and MSNs). Additionally, we constructed a 6-feature network using the T1w/T2w ratio as an additional feature. T1w/T2w was computed by first registering the T2w image to the processed T1w image, then projecting both the T1- and T2-weighted images to the cortical surface using Freesurfer's `mris_vol2surf` command and taking the vertex-level ratio. The resulting 6-feature network was highly similar to the original five-feature network with an edgewise correlation between them of $r = 0.78$. From these results, we conclude that while structural similarity is influenced by feature selection to some extent as expected, the overall network structure does not hinge disproportionately on specific features.

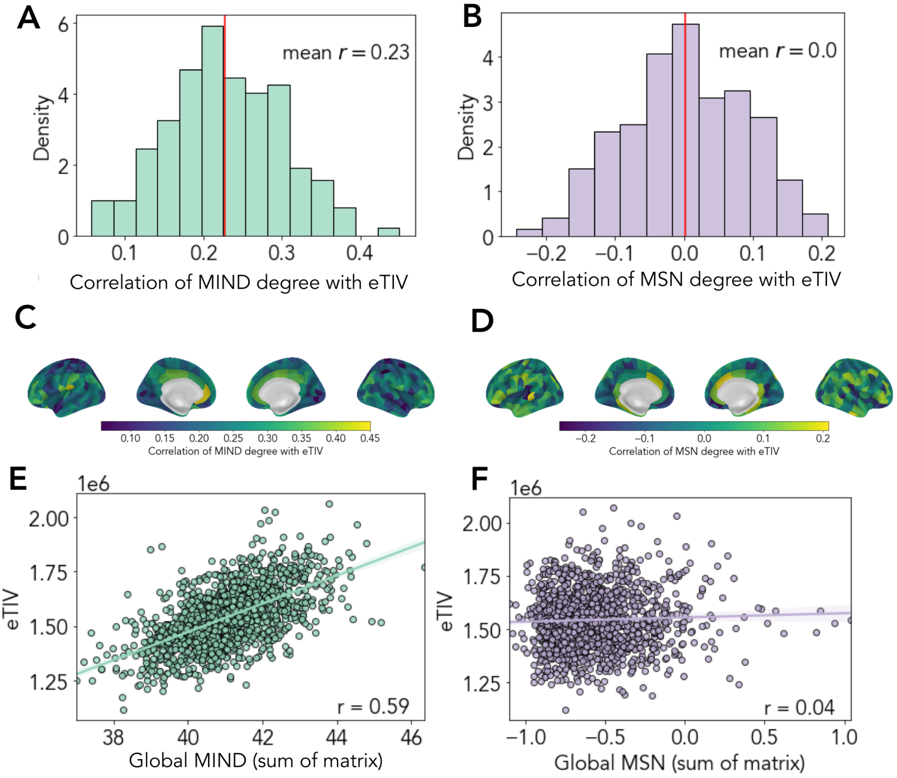

Fig. S7 Relationship between MIND, MS, and eTIV. A-B) The distribution of correlations of weighted nodal degree for MIND networks and MSNs with estimated Total Intracranial Volume (eTIV). C-D) A visualization of the spatial organization of the correlations reported in C and D on the cortex. E-F) the correlation between eTIV and global MIND and MS. Shading represents 95% CI of a best-fit line. Global network measures were calculated as the sum over the entire similarity matrix. These global values are strongly related to eTIV in MIND networks ($r = 0.59$), but not in MSNs ($r = 0.04$). In future work, these relationships will be important to consider when interpreting results – for example, by ensuring that regional differences in MIND observed in a case-control study are not due exclusively to differences in brain size.

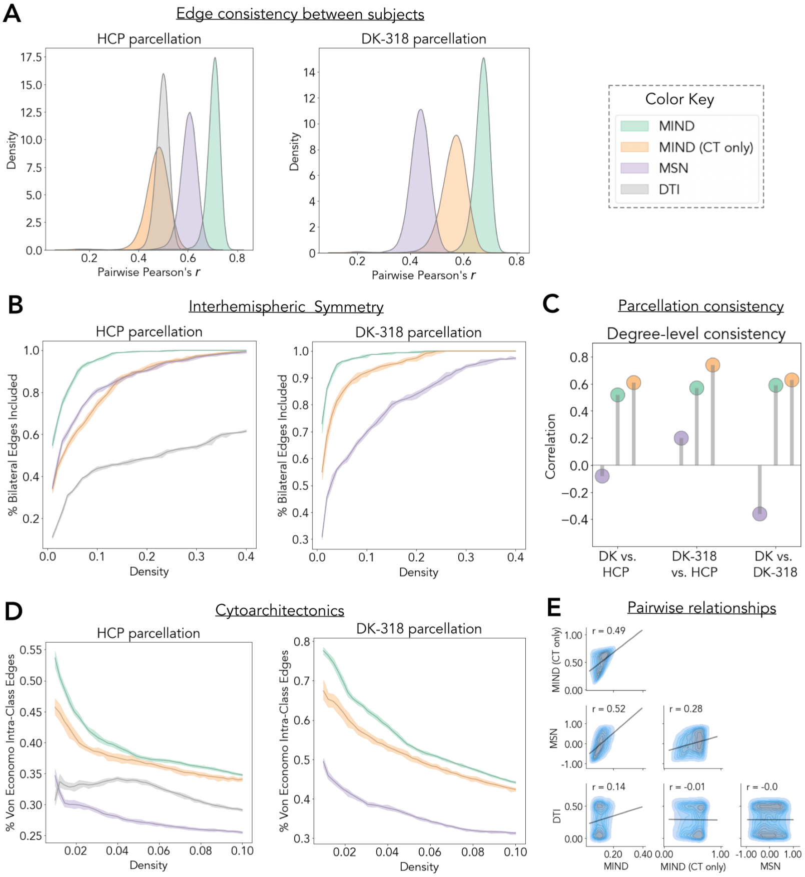

Fig. S8 Comparing multivariate MIND networks, MSNs, DTI networks and univariate MIND networks in the HCP-YA cohort. We replicated and extended the main findings from Figure 2 in the HCP-YA dataset, additionally comparing to individual DTI connectomes and univariate MIND networks computed using vertex-level estimates of cortical thickness alone. A) The distribution of pairwise correlations of network edges between subjects for MIND networks, MSNs, DTI networks, and univariate CT-based MIND networks - indicated as MIND (CT-only) – for all pairs of subjects in the HCP-YA cohort. B) The fraction of total inter-hemispheric connections represented at different network densities for all types of group mean networks. C) Parcellation consistency of a nodal phenotype (weighted degree) in multivariate MIND networks and MSNs, and univariate MIND networks, using the method described in Figure 2. D) The fraction of edges between regional nodes of the same cytoarchitectonic class over a range of network densities. E) The pairwise relationship between edges of the group-average MIND network, univariate CT-based MIND network, MSN, and DTI connectome. In panels B and D, shading represents a 95% CI determined by population bootstrapping (solid line represents the mean over all bootstraps). In panels A and D, MIND networks and MSNs are shown in either the HCP (360 region) or DK-318 (318 regions) parcellations, whereas DTI networks are only shown for the HCP parcellation due to data availability.

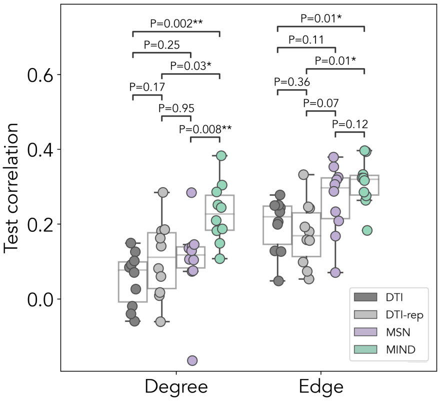

Fig. S9 Replicating HCP-YA age prediction comparison. An analogous plot to those shown in Figure 4, comparing the performance of models trained to predict age using node degree or edge weights of MIND networks, MSNs, or two versions of DTI connectomes in the HCP-YA cohort. The training and evaluation procedures were identical to those described in Figure 4. DTI-rep indicates the performance of predictive models trained on DTI connectivity features using the replication DTI dataset, processed separately by Rosen et al. (2017). P-values were uncorrected from a two-sided paired t-test, $*0.01 < P < 0.05$, $**0.001 < P < 0.01$. Boxplots indicate data quartiles and whiskers indicate the full data range, excluding outliers.

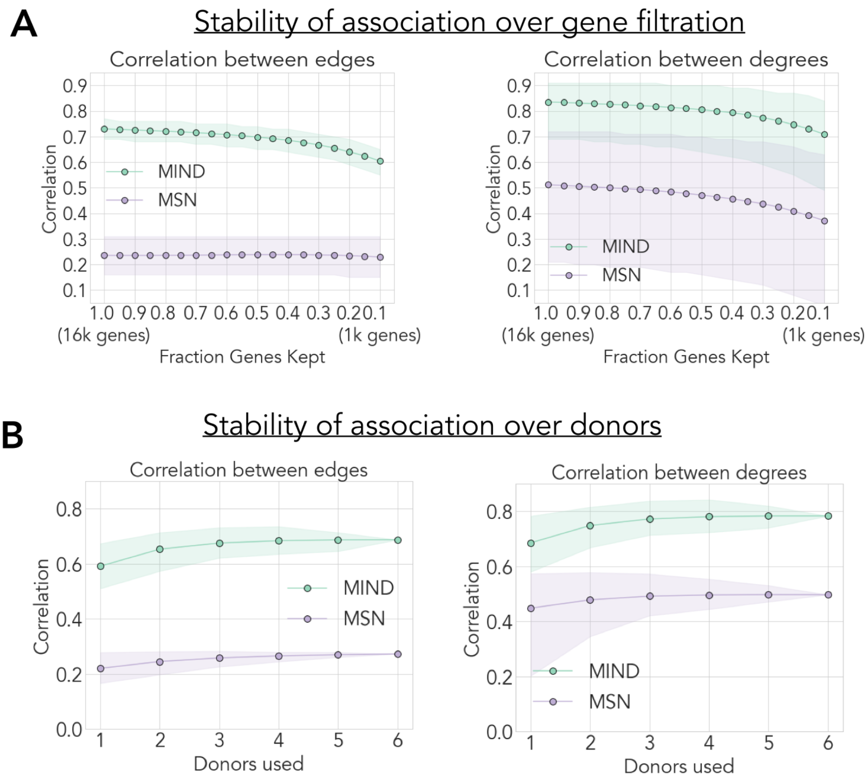

Fig. S10 Stability of association of structural similarity and gene coexpression networks. A) Stability of the correlation between structural and transcriptomic networks over genes included. Gene networks were constructed using genes filtered based on differential stability (DS) at a range of thresholds, from 100% to 10% inclusion, in 5% increments. Shading represents 95% CI. As shown, the correlation between structural similarity and transcriptomic similarity was highly conserved across the full set of filtering steps, even in the most restrictive case which included only the most stable 10% of genes. B) Stability of the correlation between structural and transcriptomic networks over AHBA donors included. The first plot is replicated from Figure 5D for ease of comparison. For each number of donors included, all combinations of gene similarity networks were constructed using the set of 29 regions covered by all 6 donors (with no gene filtering) and the mean edge and degree correlation were calculated. There were $\binom{6}{n}$ possible networks created for n included donors. Shading indicates the minimum and maximum value of the association observed at each number of included donors. The pattern of correspondence was highly stable, with a slight and monotonic gain as more donors were included, reflecting a gene-structure connection that becomes stronger as the signal to noise ratio improves. Interestingly, even when considering gene similarity networks generated from each donor independently, the variance of the correlation with MIND was relatively low. Thus, despite the small sample size from the AHBA, these results point to a shared and replicable structural and transcriptomic cortical architecture in the general population.

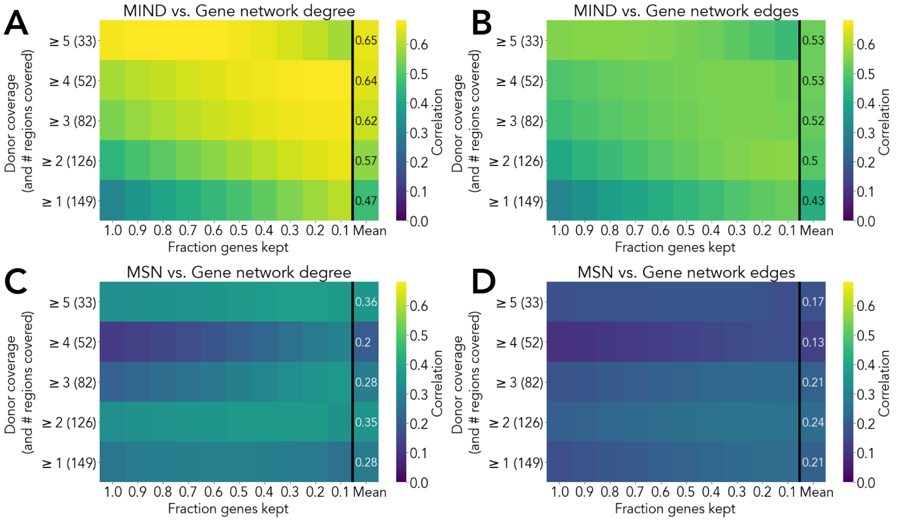

Fig. S11 Correspondence between structural and transcriptomic similarity networks, alternate parcellation. Heatmaps showing structural vs. transcriptomic degree (A, C) and edge (B, D) correlations using the DK-318 parcellation. Gene networks were constructed over a grid of gene filtering (100%- 10% inclusion using DS) and regional filtering ($\geq$1-donor coverage to $\geq$5-donor coverage) steps. Structural networks were associated at each combination of filtration steps. The rightmost column of each heatmap reports the mean association at each level of regional filtering. As only 6 regions (3.8%) were covered by all 6 donors, we did not consider filtering only for regions with complete 6-donor coverage. Overall, at both the degree and edge level when using the DK-318 parcellation, the original result is reproduced that MIND networks demonstrate much higher correspondence than MSNs with the gene similarity networks.

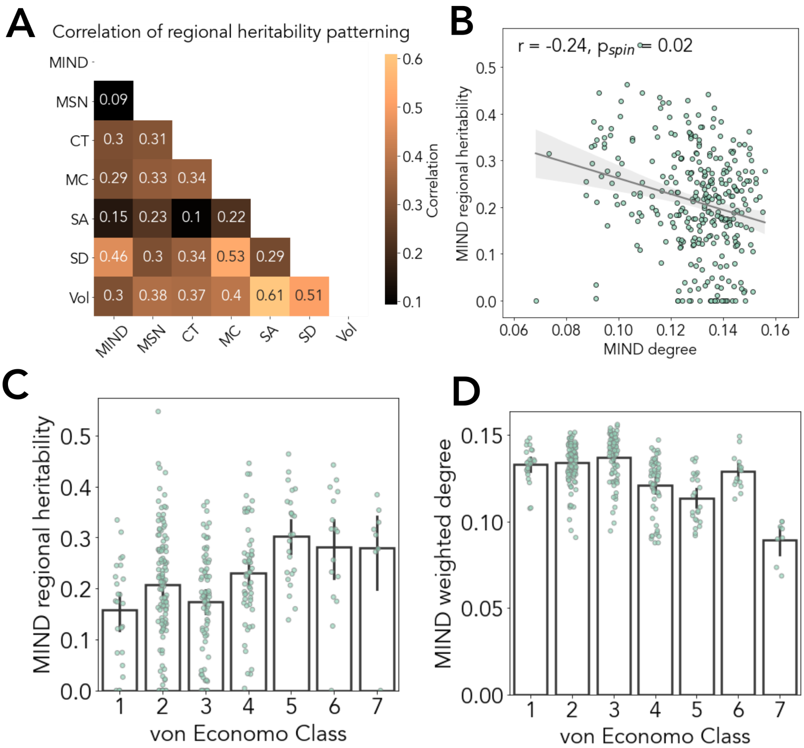

Fig. S12 Characterizing patterns of MIND heritability. A) Correlation matrix of the relationship between heritability patterns across the cortex for all studied regional features, showing a positive correlation between all vectors of regional heritabilities. B) Scatterplot of h^2_{twin} versus MIND network weighted degree (N=318 regions), with fitted line indicating significant negative correlation; each point is a node in the network. This is consistent with the negative correlation between MIND edge strength and h^2_{twin} observed in Fig. 6. The reported P-value was uncorrected and based on a two-sided spin test. Shading represents 95% CI of a line of best fit. C) Twin-based heritability of MIND network weighted degree (N=318 regions) by different cytoarchitectonic (von Economo) classes: 1, agranular cortex, primary motor cortex; 2, association cortex; 3, association cortex; 4, secondary sensory cortex; 5, primary sensory cortex; 6, limbic regions; 7, insular cortex (Seidlitz et al., 2018). D) MIND network degree (N=318 regions) by different cytoarchitectonic (von Economo) classes. Error bars in panels C and D represent one standard error of the mean.

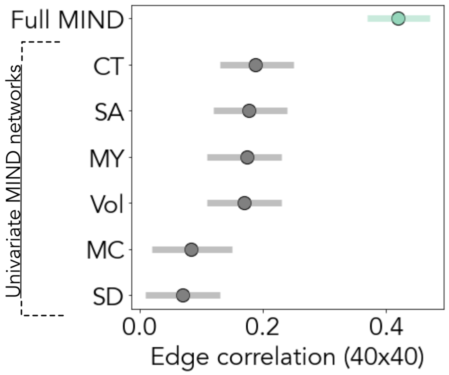

Fig. S13 Tract tracing vs. univariate and multivariate MIND networks Using the dataset of 19 macaque brain MRI scans, univariate MIND networks were constructed from each of the individual six MRI features used in the main analysis. The plot shows the correlation between the resulting network edges (N=978 edges) and tract-tracing connectivity from the Markov $\{40 \times 40\}$ parcellation, as well as for the full 6-feature MIND network using all MRI features. Shading indicates 95% CI of estimated correlations. All univariate networks were positively correlated with tract-tracing connectivity ($0.07 < r < 0.19$). However, the six-feature multivariate MIND network had greater than two-fold higher correlation with axonal connectivity estimates ($r = 0.41$) compared to the most highly-correlated univariate network, thus demonstrating one biological motivation for multimodal approaches to estimation of cortical similarity networks.

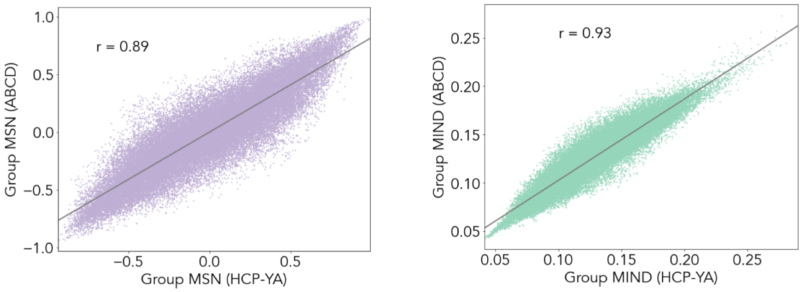

Fig. S14 Structural similarity network consistency across ABCD and HCP cohorts. The edge-wise correlation between group-level MIND networks (right panel) and MSNs (left panel) derived from the ABCD and HCP-YA cohorts (DK-318 parcellation). Both networks demonstrated high concordance between ABCD and HCP-YA group network edges ($r = 0.93$ and 0.89, respectively). Given the age difference between these cohorts (ages 9-11 vs 21-35 years of age), this consistency suggests that the structure of the cortex is largely established very early in life and indicates that relating MIND network architecture to adult-derived brain maps (e.g. of gene co-expression and cytoarchitectural class) may be valid for young and old brains alike.
