# Supplementary material for: Robust estimation of cortical similarity networks from brain MRI
Source: Nat Neurosci. 2023 Jul 17;26(8):1461–71. doi: 10.1038/s41593-023-01376-7 (PMC10400419; doi:10.1038/s41593-023-01376-7)
Supplement: Supplementary file 2 — Reporting Summary [file 41593_2023_1376_MOESM2_ESM.pdf]

nature portfolio

Corresponding author(s): Isaac Sebenius

Last updated by author(s): Jul 1, 2023

Reporting Summary

Nature Portfolio wishes to improve the reproducibility of the work that we publish. This form provides structure for consistency and transparency in reporting. For further information on Nature Portfolio policies, see our Editorial Policies and the Editorial Policy Checklist.

Statistics

For all statistical analyses, confirm that the following items are present in the figure legend, table legend, main text, or Methods section.

n/a	Confirmed	
☐	☒	The exact sample size (*n*) for each experimental group/condition, given as a discrete number and unit of measurement
☐	☒	A statement on whether measurements were taken from distinct samples or whether the same sample was measured repeatedly
☐	☒	The statistical test(s) used AND whether they are one- or two-sided<br>*Only common tests should be described solely by name; describe more complex techniques in the Methods section.*
☐	☒	A description of all covariates tested
☐	☒	A description of any assumptions or corrections, such as tests of normality and adjustment for multiple comparisons
☐	☒	A full description of the statistical parameters including central tendency (e.g. means) or other basic estimates (e.g. regression coefficient) AND variation (e.g. standard deviation) or associated estimates of uncertainty (e.g. confidence intervals)
☐	☒	For null hypothesis testing, the test statistic (e.g. *F*, *t*, *r*) with confidence intervals, effect sizes, degrees of freedom and *P* value noted<br>*Give P values as exact values whenever suitable.*
☒	☐	For Bayesian analysis, information on the choice of priors and Markov chain Monte Carlo settings
☒	☐	For hierarchical and complex designs, identification of the appropriate level for tests and full reporting of outcomes
☐	☒	Estimates of effect sizes (e.g. Cohen's *d*, Pearson's *r*), indicating how they were calculated

Our web collection on statistics for biologists contains articles on many of the points above.

Software and code

Policy information about availability of computer code

Data collection	No software was used for data collection as no novel data were collected as part of this study.
Data analysis	Python code for MIND calculation is available at https://github.com/isebenius/MIND and https://doi.org/10.5281/zenodo.7974716. FreeSurfer v5.3 was used to process T1w MRI images from the ABCD and HCP-YA datasets, v6.0 for the HCP-D dataset. The abagen v1.0.3 python package was used for Allen Human Brain Atlas analysis. We used the umx package (version 2.10.0) using R (version 4.1.3) to implement the structural equation model of the ACE model for heritability analysis. GCTA software (v1.93) was used to conduct SNP-based heritability analysis. The NeuroCombat v0.2.12 python package was used to correct site effects in the neuroimaging data. Data analysis was conducted in Python v3.6 using corresponding standard python packages including sklearn (v0.24.1), numpy (v1.17.1), scipy (v1.5.4), and pandas (v0.25.1).

For manuscripts utilizing custom algorithms or software that are central to the research but not yet described in published literature, software must be made available to editors and reviewers. We strongly encourage code deposition in a community repository (e.g. GitHub). See the Nature Portfolio guidelines for submitting code & software for further information.

Data

Policy information about availability of data

All manuscripts must include a data availability statement. This statement should provide the following information, where applicable:

- Accession codes, unique identifiers, or web links for publicly available datasets
- A description of any restrictions on data availability
- For clinical datasets or third party data, please ensure that the statement adheres to our policy

The preprocessed macaque data can be accessed at https://balsa. wustl.edu/reference/976nz. Tract-tracing connectomes based on the Markov parcellation can be accessed through https://core-nets.org. The multimodal connectome using the RM parcellation (as well as the RM atlas itself) can be accessed at https:// zenodo.org/record/1471588#.YqBt5S2ca U. Data from the ABCD cohort requires access to the NIMH data archive (NDA) and can be applied for at https:// nda.nih.gov/abcd. Our work is registered as study #1796 on the NIMH data archive, DOI 10.15154/1528079. HCP-YA data can be accessed and downloaded at https://www.humanconnectome.org. Individual DTI connectomes provided by Arnatkevičiūtė et al. (2020) for the HCP-YA dataset can be downloaded at https:// zenodo.org/record/4733297# .Y8wVoS-l368. HCP-YA connectomes used for replication, processed by Rosen et al. (2021), can be accessed at https://zenodo.org/ record/4060485#.Y858GS-l0Q0. HCP-Development data can be accessed and downloaded by following the instructions at https://www.humanconnectome.org/ study/ hcp-lifespan-development/data-releases. Consensus HCP-YA DTI connectivity in DK parcellation can be downloaded directly from the ENIGMA toolbox at https://enigma-toolbox.readthedocs.io/en/latest/. Expression data from the Allen Human Brain Atlas can be downloaded using the abagen package at https:// abagen.readthedocs.io/en/stable/.

Human research participants

Policy information about studies involving human research participants and Sex and Gender in Research.

Reporting on sex and gender	Data on biological sex for the ABCD cohort was determined by the demographic data received from the NIMH data archive. Data on biological sex for the HCP-YA and HCD-D cohorts was determined based on the demographic data provided by the publicly available metadata. Sex was not considered in any of the analyses concerning group-level networks, which we constructed using data from both male and female subjects. For the SNP-based heritability analysis, biological sex was included (alongside age*sex and age^2*sex) as a covariate. In the twin-based analyses, all dizygotic twins were sex-matched, hence sex was not further considered here. All macaque data were derived from female macaques. For the age prediction analyses, sex was not considered during the training phase, but was included as a covariate (binarized as 0/1) when evaluating model performance.
Population characteristics	The participants in the ABCD cohort, at the baseline scan sessions we considered, were between 9-11 years of age (48% female). The data were collected to form a representative, diverse population sample to study longitudinal brain and cognitive development, and hence reflected a wide range of ethnicities. Diagnostic information about the population were not considered, although the ABCD cohort includes subjects with diverse neurodevelopmental profiles. Further details on ABCD population characteristics can be found in Garavan et al., 2018.<br><br>We additionally used data from the HCP-Development (HCP-D) cohort (N=655, aged 8-21, 49% male), and the HCP-Young Adult cohort (N=960, aged 21-35, 46% male).
Recruitment	No participants were recruited for this study – we only used publicly available data.
Ethics oversight	This study used only publicly available data. Approval for use of the ABCD data fell under an NDA agreement, reflected in study #1796 on the NDA website.

Note that full information on the approval of the study protocol must also be provided in the manuscript.

Field-specific reporting

Please select the one below that is the best fit for your research. If you are not sure, read the appropriate sections before making your selection.

☒ Life sciences ☐ Behavioural & social sciences ☐ Ecological, evolutionary & environmental sciences

For a reference copy of the document with all sections, see nature.com/documents/nr-reporting-summary-flat.pdf

Life sciences study design

All studies must disclose on these points even when the disclosure is negative.

Sample size	11,449 subjects from the ABCD cohort were available with neuroimaging data, of which 10,367 were used as the primary cohort (for the analyses based on the DK-318 parcellation). 1,282 twin subjects (641 pairs) were used for the twin based analyses, and 4,085 (unrelated) subjects of primarily European ancestry were used for SNP-based heritability analyses. Of these two sub-cohorts, 432 subjects overlapped. We additionally used data from the HCP-Development (HCP-D) cohort (N=655, aged 8-21, 49% male), and the HCP-Young Adult cohort (N=960, aged 21-35, 46% male). Sample sizes were not predetermined; we used all available samples that met the inclusion criteria. For our analyses, these sample sizes were sufficient as evidenced for example by a) the high correlation between group networks across ABCD (N>11,000) and

HCP-YA (N=960) cohorts and b) the replication of the age prediction results across HCP-YA (N=960) and HCP-D (N=655) cohorts. Moreover, the sample sizes used for twin heritability and SNP heritability analysis were comparable to the sample sizes used in recent studies using ABCD to study the heritability (Bethlehem et al., 2022) and genetics (Warrier et al., 2022) of brain structure.

Data exclusions

Data from the ABCD cohort were excluded based on two reasons: if they had poor quality scans (Euler index below -120), or if any regions in the DK-318 parcellation were not assigned any vertices in the cortical surface reconstruction. For the twin-based heritability analyses, triplets were excluded. For the SNP heritability analyses, subjects were excluded if they failed to meet the genetic quality control criteria: if their genotyping rate was less than 95%, if their genetic sex did not match their reported sex, or if they were determined not to be of primarily European genetic ancestry, measured using multidimensional scaling after including subjects from the 1000 Genomes phase 3 data. For the HCP-YA and HCP-Development cohorts, subjects were excluded if they did not have corresponding DTI connectivity data as published by Arnatkevičiūtė et al (2020) or Rosen et al (2021).

Replication

Generalization of the main findings was tested based on extensive sensitivity analyses. The main finding regarding the Allen Human Brain Atlas was replicated using each of the six individual donor brains separately, rather than aggregating across all donors. Cross-cohort replication of the major findings from the ABCD cohort was achieved in the HCP-YA cohort.

Randomization

No data were collected in this study and no experimental groups were constructed in this study.

Blinding

There were no group comparisons in our study, nor was it an interventional study – hence no blinding was necessary.

Reporting for specific materials, systems and methods

We require information from authors about some types of materials, experimental systems and methods used in many studies. Here, indicate whether each material, system or method listed is relevant to your study. If you are not sure if a list item applies to your research, read the appropriate section before selecting a response.

Materials & experimental systems

n/a	Involved in the study
☒	☐ Antibodies
☒	☐ Eukaryotic cell lines
☒	☐ Palaeontology and archaeology
☒	☐ Animals and other organisms
☒	☐ Clinical data
☒	☐ Dual use research of concern

Methods

n/a	Involved in the study
☒	☐ ChIP-seq
☒	☐ Flow cytometry
☐	☒ MRI-based neuroimaging

Magnetic resonance imaging

Experimental design

Design type

Publicly available structural data (T1w for ABCD, HCP-YA, and HCP-Development, T1w/T2w for macaque data, and preprocessed DTI connectomes for HCP-YA data) alone were used, hence no design type was applicable.

Design specifications

Only structural images were used, so no design specifications were needed.

Behavioral performance measures

No performance measures were taken.

Acquisition

Imaging type(s)

Structural (T1w for ABCD, HCP-YA and HCP-D datasets, and T1w/T2w for macaque data) and preprocessed diffusion (HCP-YA)

Field strength

3T

Sequence & imaging parameters

Human data: T1-weighted images were 1 mm isotropic, EPI, RF-spoiled gradient echo using prospective motion correction if available, and from one of three (3T) scanner models: Siemens (Prisma VE11B-C), Philips (Achieva dStream, Ingenia), or GE (MR750, DV25-26). Matrix size 256x256, flip angle 8° for all scanners. Field of view (FOV) 256x256 for Siemens and GE scanners, 256x240 for Philips scanner.

Macaque data: The animals were anesthetized and scanned on a Siemens Skyra 3T MRI with a 4-channel clamshell coil with 0.3 isotropic resolution (T1 images: TR = 2500ms, T2 images: TR=3000ms). Flip angle: 7°. Other relevant scanning sequence data were not reported in the public release of the data we used.

HCP-Development: The data used in this work were part of Release 1.0, containing cross-sectional images (preprocessed using FreeSurfer version 6.0) from 655 subjects aged 8-21 (49% male). All 3T images were acquired on a Siemens Prisma scanner 80 mT/m gradient coil, multi-echo, and with 0.8 mm isotropic resolution. Full imaging acquisition parameters are described in detail in Harms et al. [38] and Somerville et al. [70].

HCP-Young Adult: We used data from the HCP-1200 release of the HCP-Young Adult (HCP-YA) cohort. This release provides cross sectional 3T images (preprocessed using FreeSurfer version 5.3.0-HCP as described in Glasser et al. [33]) from 1113 young adults ages 21-35. Images were 0.7mm isotropic, FOV 224x224 mm, TI=1000ms, TR=2400 ms, flip angle 8 degrees.

Area of acquisition: Whole brain

Diffusion MRI ☒ Used ☐ Not used

Parameters: Connectivity based on diffusion tractography were published by Arnatkevičiūtė et al. (2020). Diffusion images were Spin echo EPI, TR=5520 ms, TE=89.5 ms, flip angle 78 degrees, FOV 210x180, b-values 1000, 2000, and 3000 s/mm2. Detailed preprocessing steps are provided in the original publication; in summary, processing of DWI images were performed by Arnatkevičiūtė et al. (2020) using MRtrix3 [73], FSL with FMRIB Software Library [44], iFOD2 [72], and Anatomical Constrained Tractography (ACT). Connectivity strengths were based on the mean fractional anisotropy within the voxels of streamlines between cortical areas.

Preprocessing

Preprocessing software: FreeSurfer v5.3 was used to preprocess images from HCP-YA and ABCD cohorts, and v6.0 for the HCP-D cohort. The recon-all command was applied to the raw T1w nifti files with default parameters. All diffusion data was published as derived connectivity matrices; we performed no preprocessing on this data.

Normalization: Normalization steps were defined by the default recon-all pipeline from FreeSurfer v5.3. These included linear and non-linear registration to the fsaverage template and intensity normalization.

Normalization template: The fsaverage (MNI305) template was used.

Noise and artifact removal: Euler index was used as a measure of image quality, but was not regressed from data. Rather, data below a threshold of -120 were excluded.

Volume censoring: Volume censoring was not applied to structural data.

Statistical modeling & inference

Model type and settings: The only modelling and statistical tests were post-hoc analyses of derived MIND networks - voxel or cluster based analyses were not considered. Linear models did not apply.

Effect(s) tested: No effects were tested in the experimental design - only structural data were considered.

Specify type of analysis: ☐ Whole brain ☐ ROI-based ☒ Both

Anatomical location(s): Locations were based on three predefined parcellations – the Desikan Killiany (DK), DK-318, and HCP parcellations described in the main text.

Statistic type for inference (See Eklund et al. 2016): No voxel-wise or cluster-wise analyses were performed.

Correction: FDR correction and Bonferroni correction were used to correct for the main analytical results, but no correction related to voxel-wise or cluster-wise brain activation applies to this study.

Models & analysis

n/a	Involved in the study
☒	☐ Functional and/or effective connectivity
☐	☒ Graph analysis
☐	☒ Multivariate modeling or predictive analysis

Graph analysis: The connectivity measures used were network edge weights derived from MIND networks, MSNs, or DTI-derived connectivity networks (each entry in the region-by-region structural similarity matrices), or the weighted nodal degreed calculated as the sum (or equivalently, the average) of all edges connected to each regional node.

Multivariate modeling and predictive analysis: Any modeling was multivariate insofar as multiple structural features were included into the construction of MIND network and MSN phenotypes. To construct MIND networks and MSNs, we used vertex-level and regional estimates of grey matter volume, surface area, sulcal depth, mean curvature, and cortical thickness. To relate regional gene expression patterns to the distribution of MIND network degrees, we cross-decomposed the {1x34} matrix of MIND network degrees with the {34x15,633} matrix of regional gene expression signatures from the Allen Human Brain Atlas using partial least squares regression.

We trained machine learning models to predict the age of participants in either the HCP-D and HCP-YA cohorts using node degree or edge weights of MSNs, MIND networks, and DTI connectivity matrices. To align with the majority of the other analyses in the paper, the DK-318 parcellation was used for the HCP-D cohort, where DTI was not available. For the HCP-YA cohort, we used the HCP 360-region parcellation to match the parcellation scheme provided by Arnatkevičiūtė et al. (2020) and Rosen et al. (2021). All models were trained

on 10 train/test splits (90% train data, 10% test data) with nonoverlapping test sets. Models were implemented in Python 3.6 using sklearn. Models trained on node degree used 5-fold cross validation for each training set over a set of non-linear and linear models: specifically, a support vector machine with an RBF kernel (sklearn specification: SVR(kernel = 'rbf')) and C regularization values of 0.1, 1.0, 10, or 100, and a linear Gaussian process (GP) regression model with a summed linear and noise kernel (sklearn specification: GaussianProcessRegressor(kernel=DotProduct() + WhiteKernel(noise level bounds=(1e-10, np.inf)). The linear GP is equivalent to a Bayesian linear regression, with the noise kernel modelling the presence of i.i.d noise. All training sets were standardized using sklearn's StandardScaler() function; test sets were accordingly transformed using the normalization function estimated on the training set. For models trained on all individual edges, due to the very large number of features (> 50,000 features) we used the GP regression model alone, as in Morgan et al. (2021).

To ensure that model predictions were not biased by the presence of confounds related to subject age, to evaluate model performance we used the partial Spearman correlation of predicted versus true age, controlling for the effect of sex, Euler number (a measure of scan quality which is known to have a strong relationship with age), and a global matrix coefficient (defined as the sum over the entire connectivity matrix). This post-hoc confound adjustment ensures proper correction for the potential effect of confounds by avoiding the statistical issues that arise when regressing confounds from feature space before model training (Dinga et al. 2020).
